# Supplementary material for: Recent intensified erosion and massive sediment deposition in Tibetan Plateau rivers
Source: Nat Commun. 2024 Jan 24;15:722. doi: 10.1038/s41467-024-44982-0 (PMC10808212; doi:10.1038/s41467-024-44982-0)
Supplement: Supplementary file 1 — Supplementary Information [file 41467_2024_44982_MOESM1_ESM.pdf]

**Supplementary Information**

# Recent Intensified Erosion and Massive Sediment Deposition in the Tibetan Plateau Rivers

Jinlong Li [1], Genxu Wang [1*], Chunlin Song [1*], Shouqin Sun[1], Jiapei Ma [1], Ying Wang [1], Linmao Guo [1], Dongfeng Li [2]

[1] State Key Laboratory of Hydraulics and Mountain River Engineering, College of Water Resource and Hydropower, Sichuan University, Chengdu 610065, China

[2] Key Laboratory for Water and Sediment Sciences, Ministry of Education, College of Environmental Sciences and Engineering, Peking University, Beijing 100871, China

Correspondence: Genxu Wang (wanggx@scu.edu.cn) and Chunlin Song (songchunlin@scu.edu.cn)

The Supplementary Information includes:

Calibration and Uncertainty

Limitations and Perspectives

Figs. 1 to 18

Tables. 1 to 3

References

## Calibration and Uncertainty

Satellite imagery has been successfully used to map the distribution of suspended sediments in coastal waters and large estuaries[1–5]. However, the application of such data and methods to rivers on the Tibetan Plateau (TP) remains limited. Sediments with distinct mineralogical compositions exhibit varying absorption characteristics, resulting in differences in the spectra that are most highly correlated with suspended sediment concentration (SSC) both within and among rivers. However, the transferability of local calibrations is likely limited due to the narrow range of SSCs that are included in locally calibrated datasets, or specific sediment characteristics of certain locations[6,7]. To address changes in SSC between rivers, previous studies have utilized additional calibration development methods such as *in situ* datasets on organic content or grain size[3], or setting a threshold for river SSC[8]. The scarcity of publicly available *in situ* data poses a challenge to the development of SSC calibration models, particularly for suspended sediment particle size and river properties such as chlorophyll-a[9] and organic material[8]. As a result, we can only employ K-Means clustering, proposed by Dethier et al.,[1,7] to group rivers with similar optical characteristics and calibrate the model specifically with sufficient local *in situ* measurements. Furthermore, we can only use publicly available soil particle size data near the sampling points to evaluate the uncertainty. By utilizing these two uncertainty evaluation methods, we can ensure that the calibrated average relative error is 0.26 for each cluster, and the average relative station bias for each station is also guaranteed to be 0.24.

Furthermore, previous studies have attempted to extract surface water using Landsat images through techniques such as single-band thresholding and dual-band indexing[9,10]. However, these methods have limited applicability when applied to the TP region due to their low accuracy. We adopted a multiple water index approach and partitioned the entire TP into two regions: the Himalayan region (with the highest environmental noise) and other areas. For the Himalayan region, we utilized an altitude-based division using the water multiple index method, which resulted in an overall accuracy (OA) of 0.96 and a Kappa coefficient of 0.90, as reported by ref.[11]. However, for the single-index method (MNDWI), the OA and Kappa coefficient is only 0.88 and 0.6079, respectively. We also utilized the automated surface water

fraction method[12] to evaluate the accuracy of our water mask with the Google Earth Engine (GEE). This approach can produce a water percentage, enabling us to observe sub-pixel level changes in the mask, particularly during flood periods. Since floods can persist for varying durations ranging from hours to weeks, they may cause an overestimation of SSC overall.

In summary, our goal was to minimize the uncertainty associated with satellite-based SSC assessment by considering factors such as data quality, long-term water body changes, environment noise, and flood events, as well as the transferability of local calibration (Supplementary Figs. 12-16). However, this approach encounters some inevitable uncertainty when applied to the entire TP[7]. For instance, the model may underestimate SSC for fine particles with low-quality scores due to their low reflectance per unit mass. Conversely, a significant amount of organic matter and chlorophyll-a in rivers may result in high SSC model inferences. Nevertheless, unlike the public datasets provided by the USGS (USA) and WSC (Canada), records of parameters such as the average fraction of suspended sediment and fluvial organic matter of the TP during the sampling period are unavailable. The potential effects of these parameters on rivers remain understudied due to limited data availability. Overall, this approach and associated algorithms are currently considered the most advanced and accurate technique for developing SSC calibration models on the TP, which can reduce the uncertainty to acceptable levels.

## Limitations and Perspectives

In cold regions, comprehensive, prolonged on-site observations of environmental drivers for sediment production, erosion, and sediment transport are notably lacking. This deficiency is particularly evident in the monitoring of the entire journey of riverine sediments, from their mountainous origins to their sinks[13]. Access to the limited available records is often hampered by a combination of policy restrictions and technological barriers, resulting in sporadic and incomplete studies. Consequently, these constraints may introduce biases in research outcomes, potentially leading to underestimations of sediment production, unreported erosional reductions in specific cryospheric regions, or uncertainties stemming from satellite calibration[6,7]. These constraints impede a full assessment of landscape changes, spatiotemporal

variations in sediment dynamics, and the response of basin-scale sediment transport to climate changes.

Furthermore, the scarcity of *in situ* observations, coupled with a lack of validation and calibration data, and insufficiently detailed parameterization of critical processes (most studies rely on localized empirical equations[2,14,15]), hinders the development and application of satellite-driven sediment calibrated models[2,4]. This is especially true for physics-based models tailored to cold regions that link hydrology, hydraulic, and sediment transport, as well as macro-scale sediment transport models. Presently, there exists an incomplete understanding and evaluation of the long-term responses of erosion and sediment transport to climate-driven cryospheric variations. Globally, most rivers lack sediment monitoring, with close to 90% of them remaining unmonitored[16]. Decadal-scale sediment observations in cryospheric regions are exceedingly rare[16,17]. Breakthroughs in deciphering the relationship between surface reflectance and SSC, along with the extension of retrieval techniques beyond calibration areas, will enable the extraction of sediment-related information from previously unused satellite imagery archives[1,2]. This holds promise for filling critical scientific gaps, especially by providing data from remote regions.

Nonetheless, substantial challenges must be surmounted to achieve these objectives in the future. This includes addressing issues related to the revisit frequency and spatial resolution of satellite products[18], minimizing uncertainties arising from the extrapolation of satellite calibration models[7], and tackling the problem of accurately discriminating sediment particles of varying sizes through satellite remote sensing, especially in turbid waters[19]. The final challenge, at least in the current state of scientific advancement, remains an exceedingly formidable task[9,20]. As technology continues to advance, the development of next-generation satellites and sensors will yield data with higher resolutions and increased richness. Moreover, the application of machine learning, particularly deep learning techniques, holds the potential to enhance the processing of satellite data, ultimately improving sediment prediction accuracy. As these technologies and methods evolve, remote sensing will remain a potent tool expected to play an increasingly pivotal role in sediment transport research[6]. Future satellite missions with global coverage will facilitate the monitoring of a greater number of rivers[21], even those situated far from traditional monitoring stations. Therefore, we are committed to transparently

sharing both our algorithms and the satellite-derived sediment data for the rivers of the Tibetan Plateau. Furthermore, we advocate for enhanced data-sharing collaborations among the global scientific community and pertinent governmental bodies. These collaborative initiatives will serve as a substantial stride toward deepening our understanding of sediment erosion in cold regions.

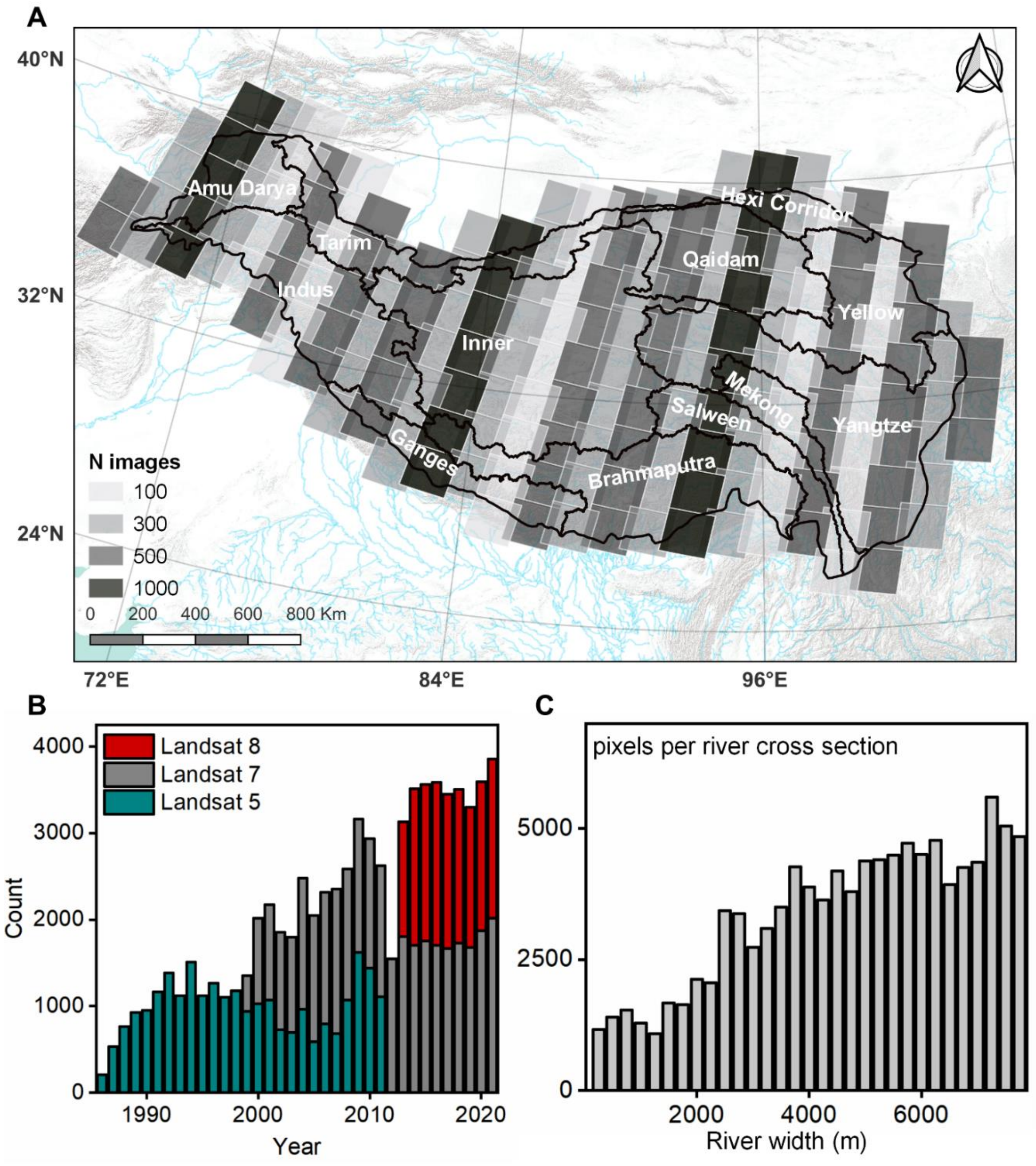


**Supplementary Fig. 1.** Summary of Landsat satellite imagery sampling. (A) The number of Landsat 5, 7, and 8 satellite samples for the Tibetan Plateau, with image strip coverage based on the number of cloud-free samples applied. Base map, inset, and Landsat shapefiles courtesy of ESRI, USGS, and NOAA (https://www.usgs.gov/landsat-missions/landsat-shapefiles-and-kml-files). (B) A histogram of the number of cloud-free images recorded by each satellite. (C) A histogram of the number of images per river cross-section, showing the average at different river widths.

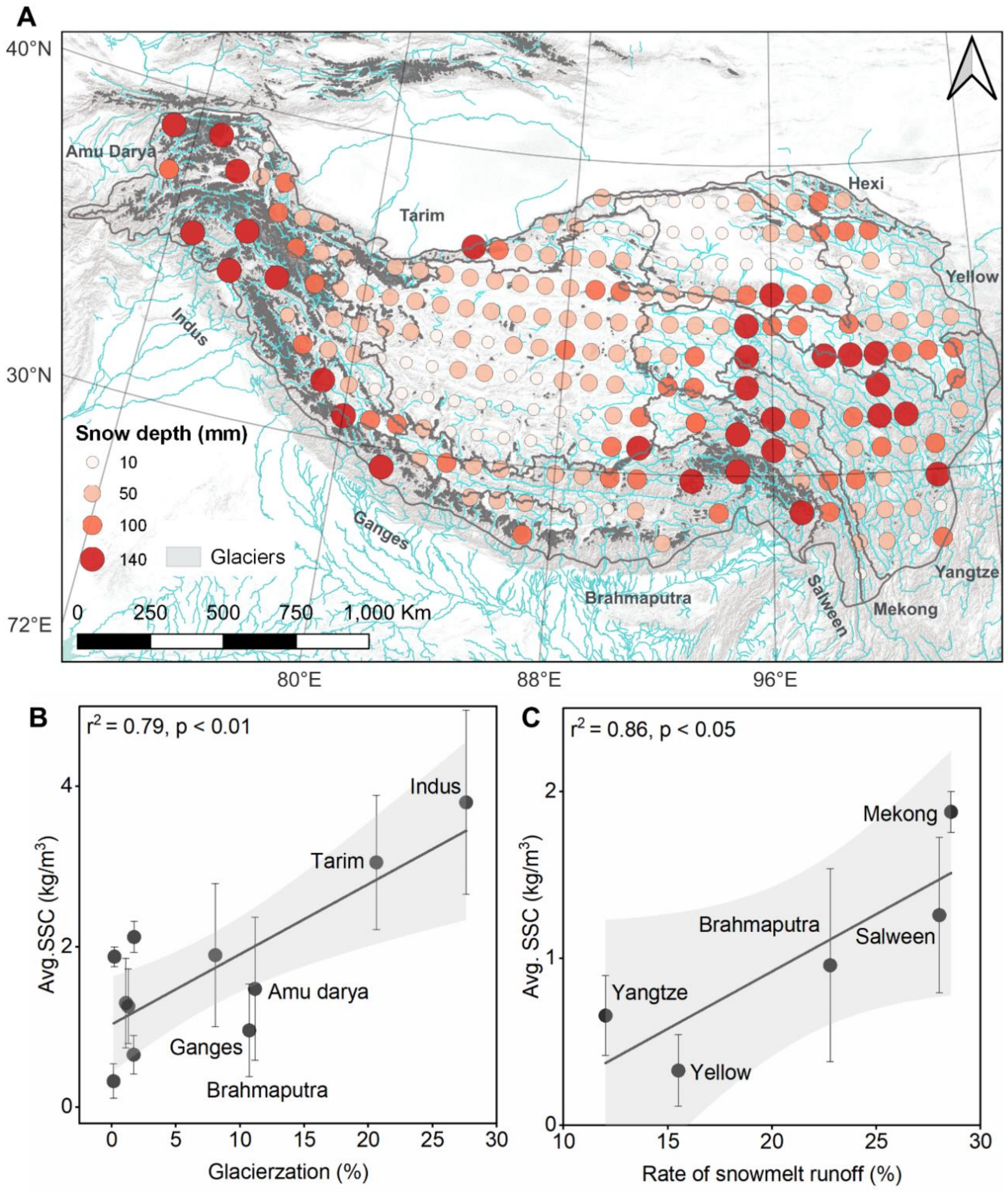


**Supplementary Fig. 2.** Impacts of glaciers and snow dynamics on suspended sediment concentration (SSC). (A) A map of annual average snow depth distribution on the Tibetan Plateau (TP). Combined with Fig. 1, it can indicate that the high-concentration sediment is concentrated in areas highly covered by glaciers and snow. Boundaries of glaciers and permafrost are based on ref[22]. Snow depth data are available on ref[23]. Base map and inset courtesy of ESRI, USGS, and NOAA. (B) Comparison of the annual average SSC with the glacierization (percentage of glacier cover for a headwater basin) of each basin, based on basin-weighted SSCs retrieved from satellite imagery. (C) The basin-weighted SSC and

snowmelt runoff ratios of the six headwater basins on the eastern TP, with annual average snowmelt runoff ratios are derived from ref[24].

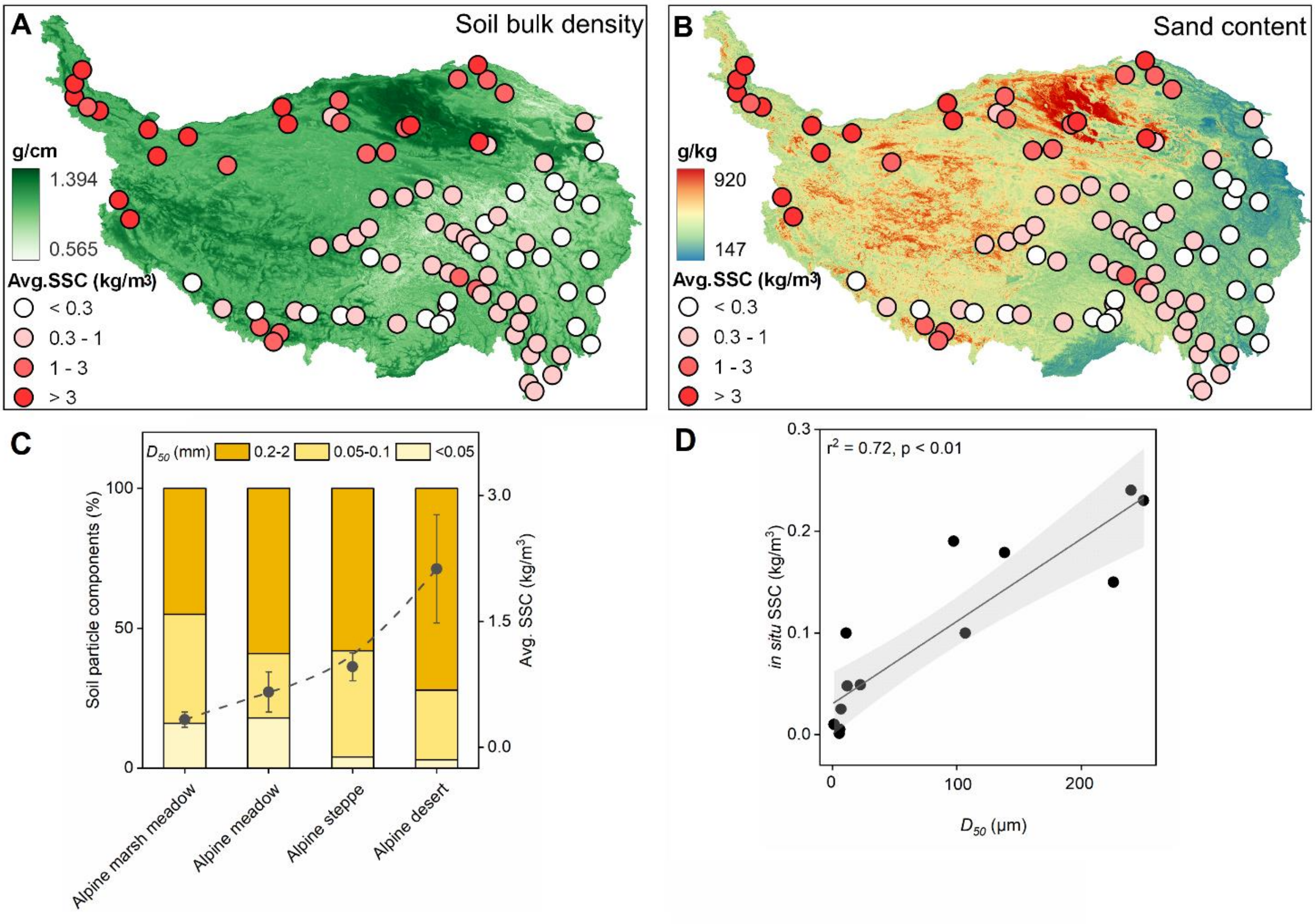


**Supplementary Fig. 3.** Impacts of soil properties on suspended sediment concentration (SSC). (A) A map of soil bulk density (0-20 cm) of the topsoil distribution on the Tibetan Plateau (TP). Soil bulk density data are sourced from refs[25]. (B) Map of sand content of the topsoil (0-20 cm) distribution. Spatially coupled with satellite-estimated SSCs, it suggests that the heterogeneity of regional soil properties directly determines the concentration of the surrounding riverine suspended sediments. Soil content data are sourced from refs[25]. (C) Comparison between soil particle composition sorting and average SSCs under the four most common vegetation cover on the TP, indicating that the coarser the soil particles and the lower the vegetation coverage, the larger river SSCs. (D) *In situ* measured SSCs increased with the increase of soil median particle size ($D_{50}$). I*n situ* measured SSCs are based on field samples by Yan et al.[26] in the headwater regions of the Yangtze River from 2012 to 2017.

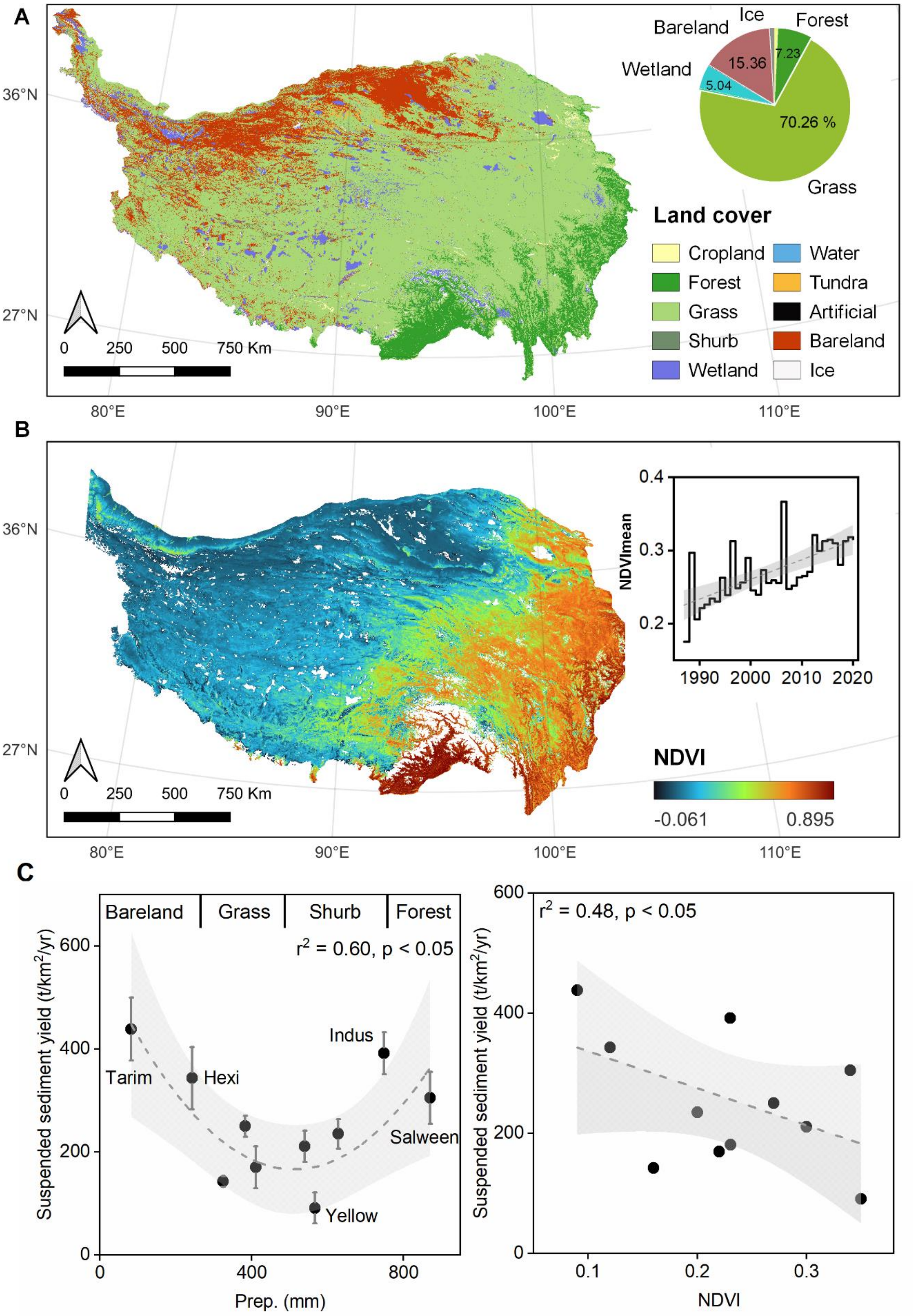


**Supplementary Fig. 4.** Spatial heterogeneity of land cover, precipitation, and vegetation cover results in varying patterns of regional sediment yield. (A) A map of land cover on the Tibetan

Plateau (TP), with a pie chart indicating the proportion of each land coverage. (B) A map of annual average Normalized Difference Vegetation Index (NDVI) on the TP, indicating an overall increase in vegetation in response to warming climate. (C) Comparison between suspended sediment yield and precipitation for each headwater basin. Land cover on the upper side is estimated from precipitation levels, revealing clear differences in east-west sediment yield patterns. The figure on the right shows an overall negative correlation between basin-weighted sediment yields and NDVI across the entire TP. Please note that the base map in (A) and (B) is only for the TP in China, due to the openness of the data. Map of Land cover and NDVI change are sourced from refs[27,28], while Prep. represents the basin-weighted precipitation calculated from ERA5 datasets[29].

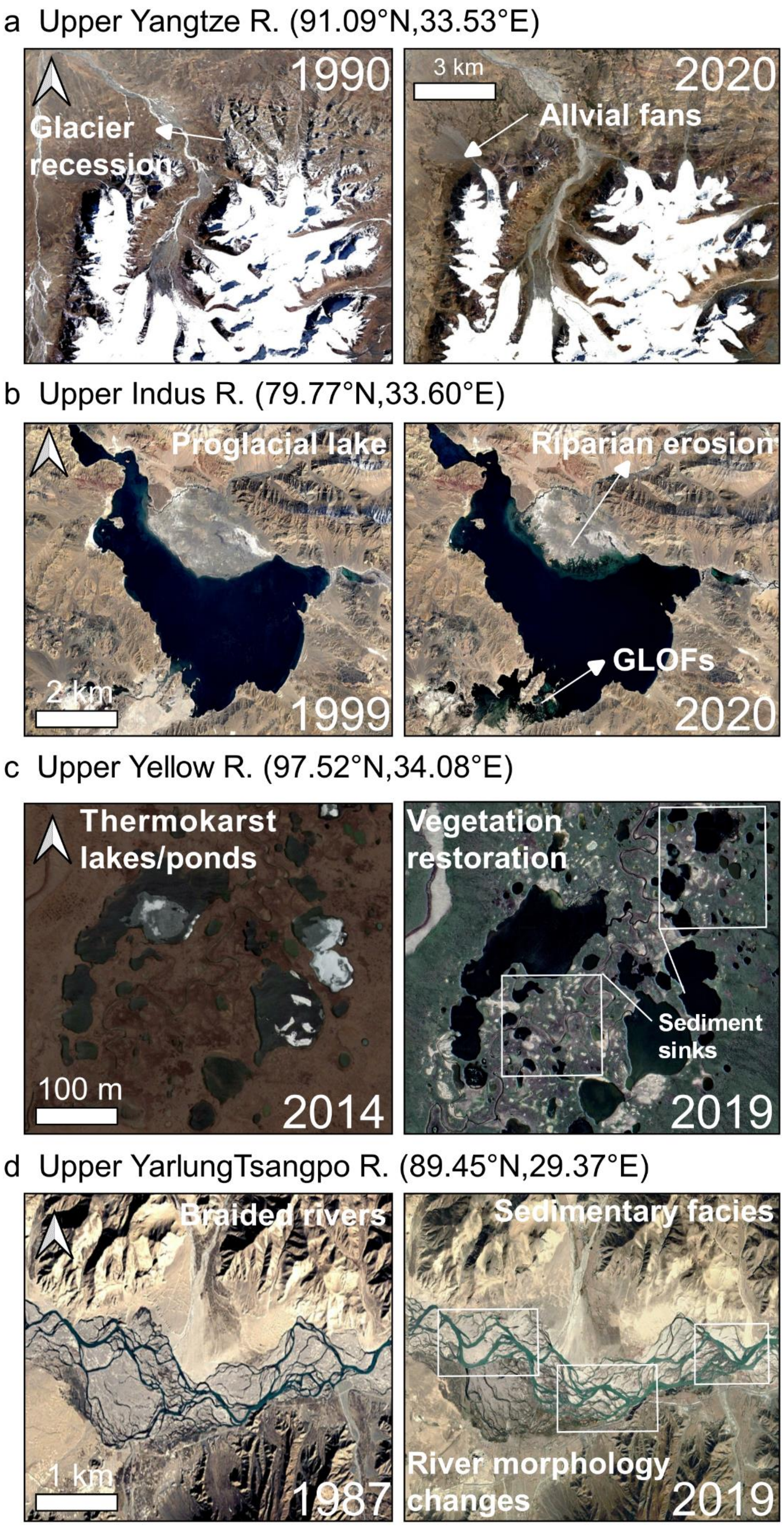
a Upper Yangtze R. (91.09°N,33.53°E)
1990
Glacier recession
3 km
2020
Allvial fans
b Upper Indus R. (79.77°N,33.60°E)
Proglacial lake
2 km
1999
Riparian erosion
GLOFs
2020
c Upper Yellow R. (97.52°N,34.08°E)
Thermokarst lakes/ponds
100 m
2014
Vegetation restoration
Sediment sinks
2019
d Upper YarlungTsangpo R. (89.45°N,29.37°E)
Braided rivers
1 km
1987
Sedimentary facies
River morphology changes
2019

**Supplementary Fig. 5.** Examples of glacier recession, thermokarst activities, and river morphology changes that drive the shifts of sediment erosion-deposition modes on affected rivers. (a) Over the past three decades, the Geladandong Glacier, located in the upper reaches of the Yangtze River source, has undergone significant retreat. As a consequence, sediment accumulation at the glacier terminus has caused the main channel to widen and lateral alluvial fans to develop, representing the continuous deposition of the majority of sediment. (b) In the upper Indus River, erosion and landslides of the shorelines of large glacial lakes have led to riverbank retreat. Additionally, downstream glacial lake outburst floods (GLOFs) have resulted in the breach of downstream lake embankments by the lake water. (c) The expansion of thermokarst lakes and the restoration of vegetation in the upper Yellow River serve as interceptors of sediment transported from the upstream area. (d) The braided channels in the middle reaches of the Yarlung Tsangpo River have undergone significant morphological changes over several decades due to continuous sedimentation and aggradation caused by upstream sediment transport. The meandering and shallow channel bed and altered channel course indicate the deposition of a large amount of sediment. Base map and inset courtesy of ESRI, USGS, and NOAA.

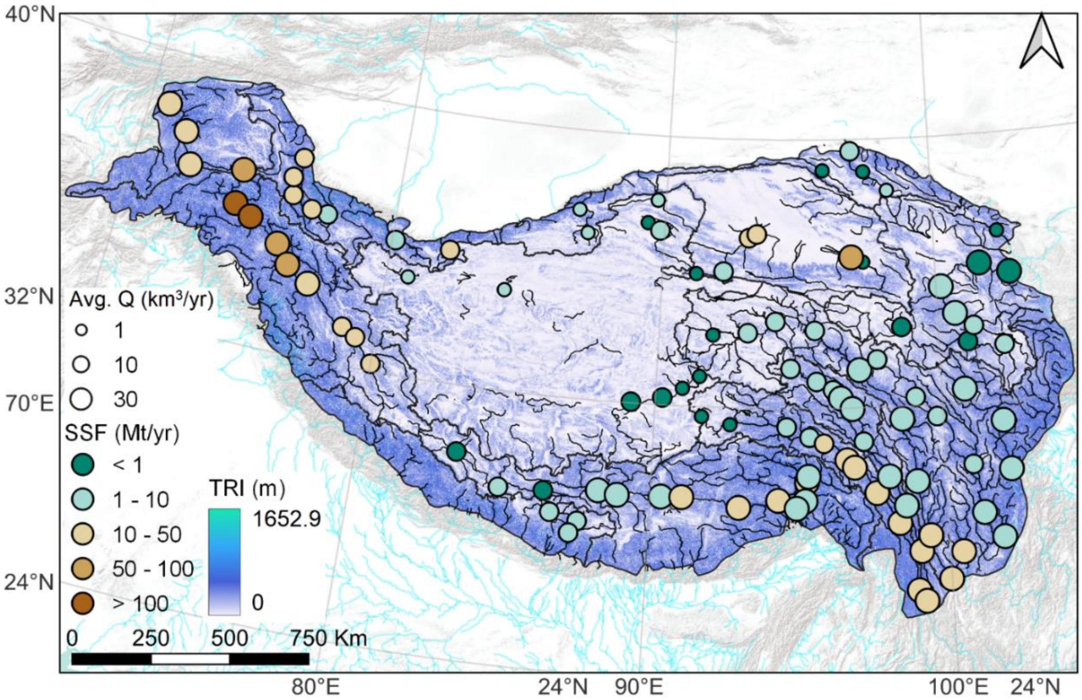


**Supplementary Fig. 6.** The runoff and sediment transport processes are affected by the topography and geomorphic patterns. The spatial variations in the terrain ruggedness index (TRI) with satellite-estimated suspended sediment flux (SSF) reveal that regions with significant elevation differences concentrate on high-flux sediment transport (indicated by brown symbols). The size of the symbols is proportional to the average runoff discharge (Q). Base map and inset courtesy of ESRI, USGS, and NOAA.

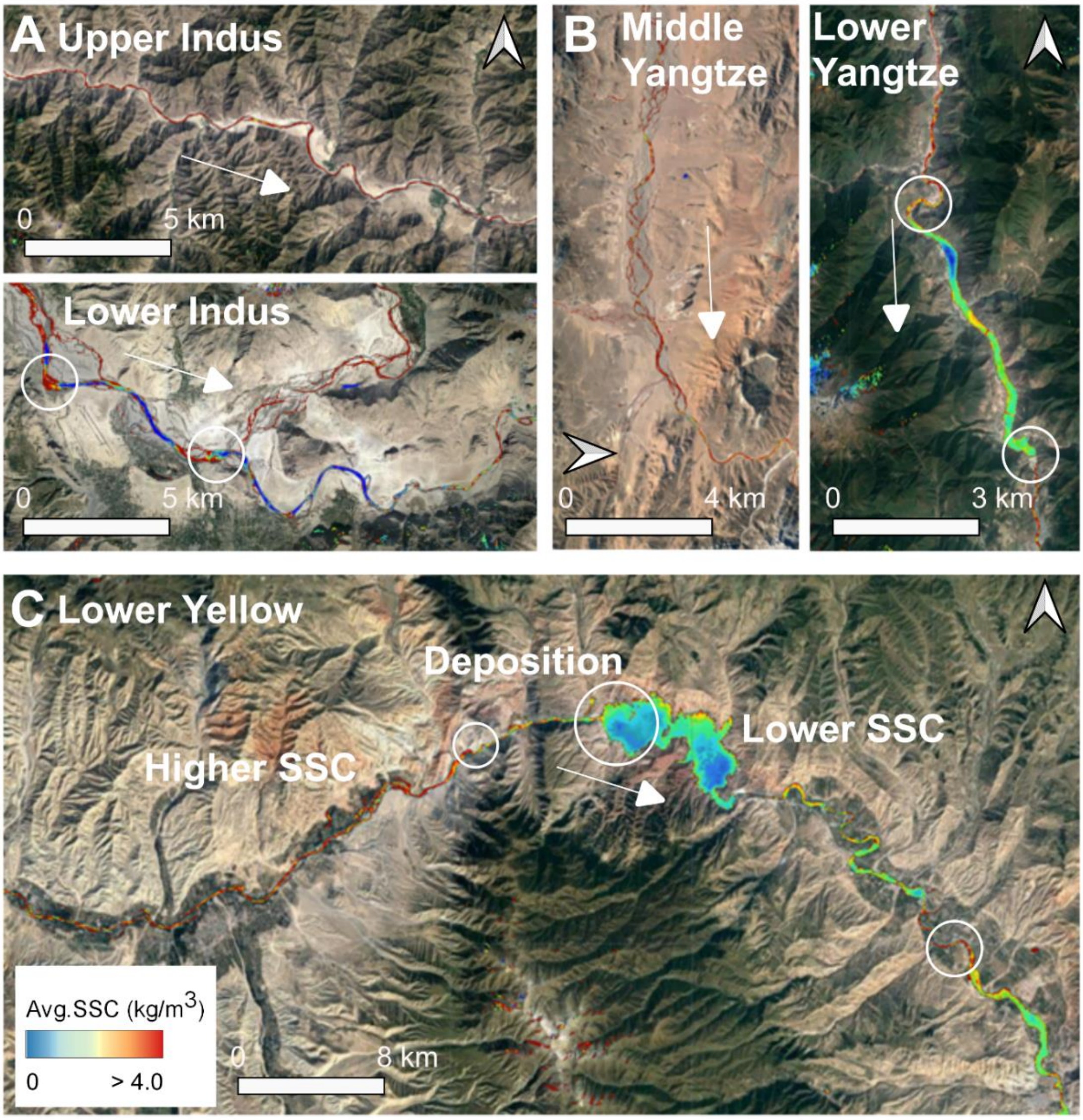


**Supplementary Fig. 7.** The transport of sediment within and between different river basins exhibits significant variations in erosion and deposition patterns. (a) In the upper Indus basin, sediment with high suspended sediment concentration (SSC) is transported downstream, originating from glacial retreat and hillslope erosion. As the Indus River reaches its downstream section, sediment erosion and deposition occur predominantly in meandering channels. (b) In the middle reaches of the Yangtze River, sediment with high SSC flows near meandering channels, while downstream, sediment with high concentration tends to deposit in narrow areas due to changes in topography and river morphology. (c) In the lower reaches of the Yellow River, lakes serve as sediment sinks, where sediment with high SSC from upstream is deposited, giving rise to significant spatial differences in sediment transport. It is noteworthy that the presented figure was generated directly by algorithms on the GEE platform, without any secondary processing. The circles in the figure represent nodes of sediment deposition, which can occur repeatedly hundreds or thousands of times in the same river. Base map and inset courtesy of ESRI, USGS, and NOAA.

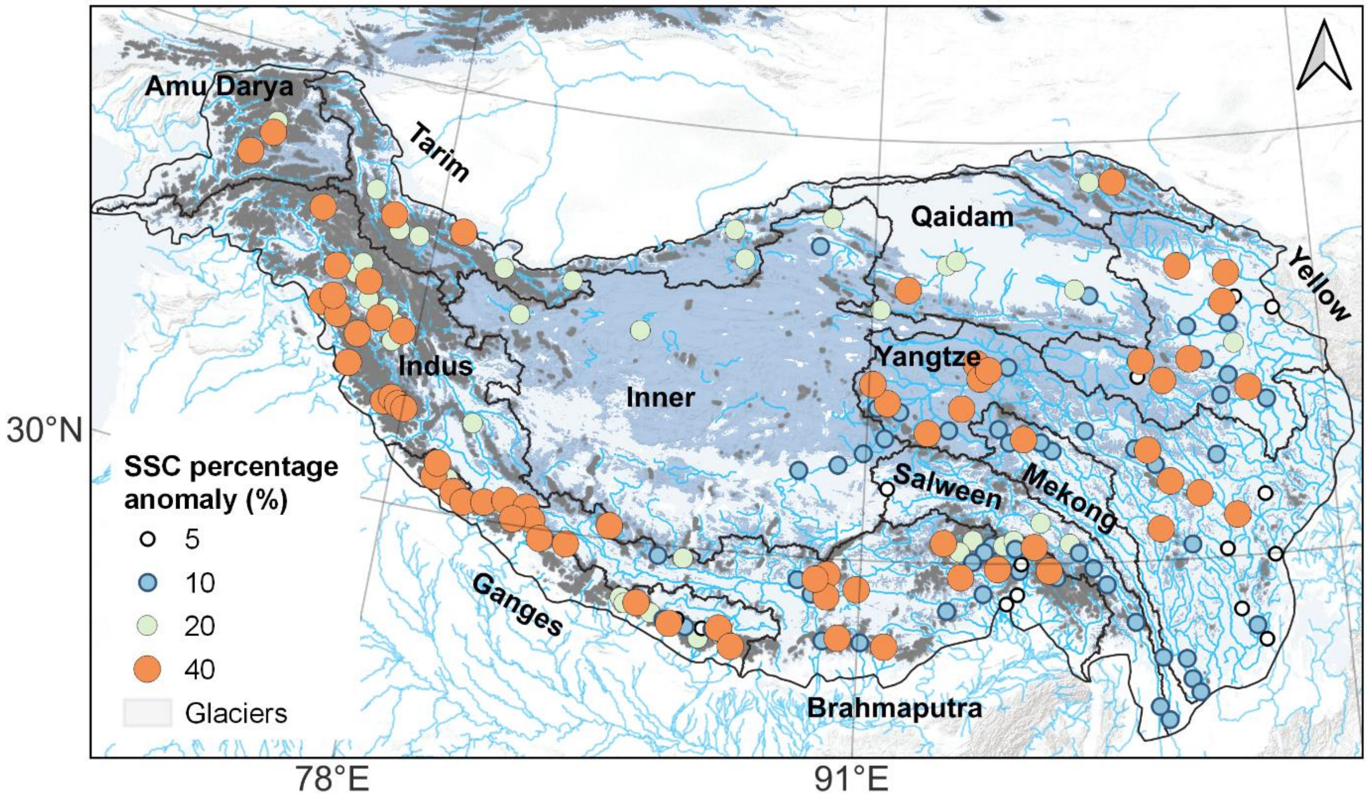


**Supplementary Fig. 8.** A catalogue of deposition locations that have experienced or have the potential to experience sedimentation. We used the SSC percentage anomaly to identify sediment deposition, which was defined as a significant increase in suspended sediment concentration (SSC) exceeding the annual mean SSC. Because the glaciers or snow likely cause environmental noise, we incorporate data on GLOF (glacial lake outburst flood) events from ref[30,31] to provide additional context. These sediment traps have the capacity to accumulate vast quantities of sediment, which can be rapidly mobilized in the event of extreme rainfall, snowmelt, or dam failure, posing a significant threat to both local ecology and hydroelectric projects in development. Base map and inset courtesy of ESRI, USGS, and NOAA.

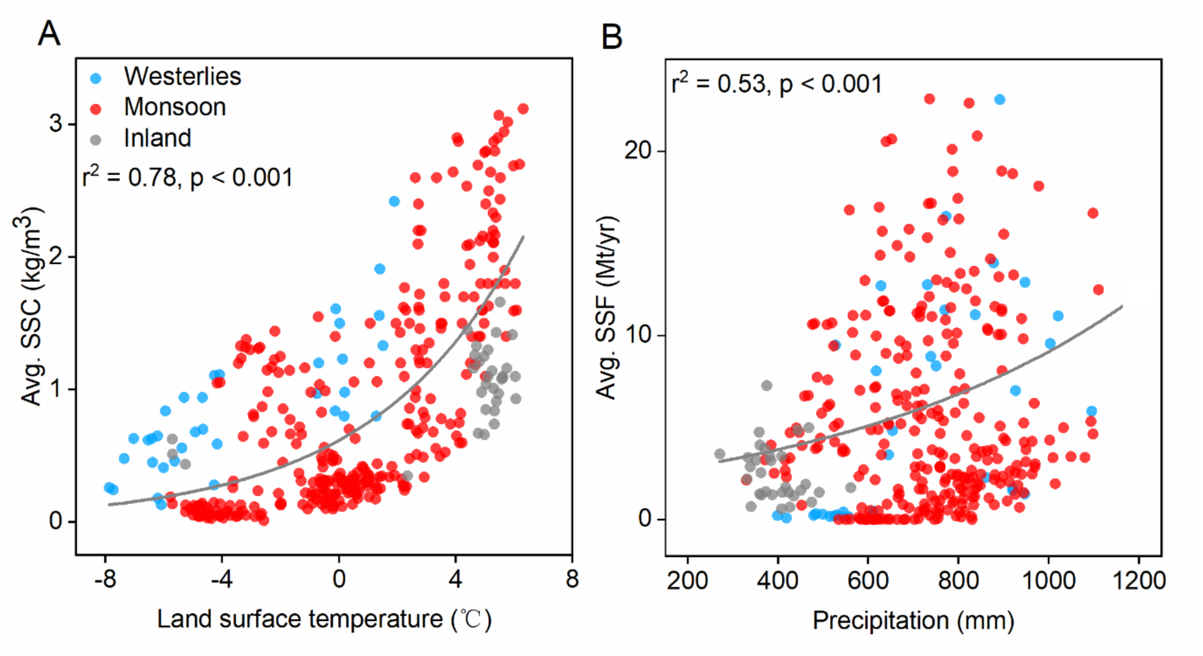


**Supplementary Fig. 9.** Satellite-derived estimation of sediment dynamics response to climate change on the Tibetan Plateau (TP). (A) The correlation between daily land surface temperature (LST) and suspended sediment concentrations (SSCs). The SSCs in the river systems of each headwater basin exhibited a positive trend with increasing LST. (B) The association between annual average precipitation and sediment source fluxes (SSFs). It is important to note that extreme rainstorms or melting events can cause a sharp and transient increase in both SSC and flux, exceeding the trend line.

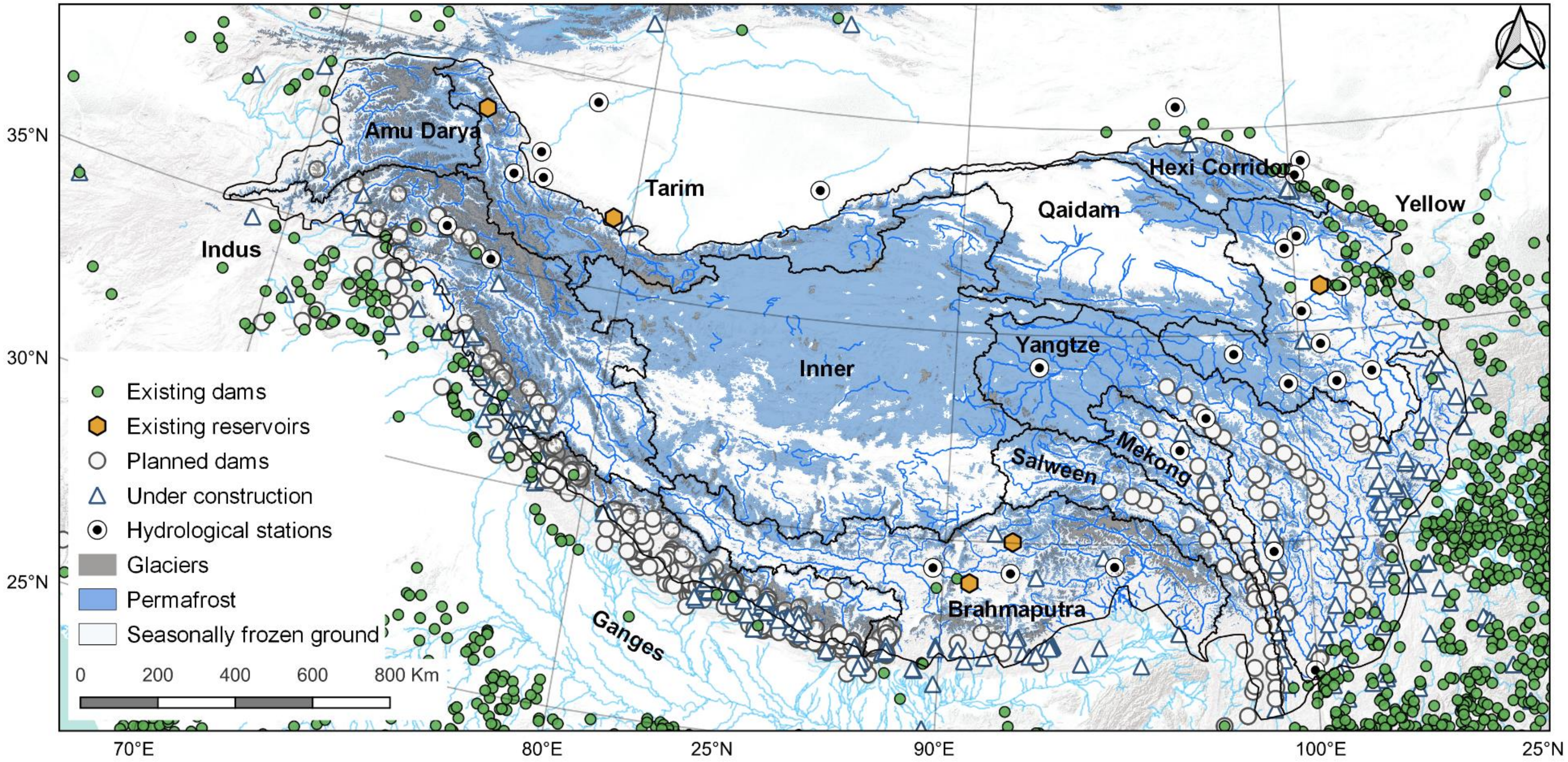


**Supplementary Fig. 10.** Existing and planned large hydropower projects, dams, and reservoirs on the Tibetan Plateau. Dams and reservoirs are from refs[32], and the planned and constructed hydropower projects are from refs[30]. Boundaries of glaciers, permafrost, and seasonally frozen ground are based on refs[33,34]. Base map and inset courtesy of ESRI and USGS. We carefully use this base map to capture sediment dynamics and minimize the impact of human activities.

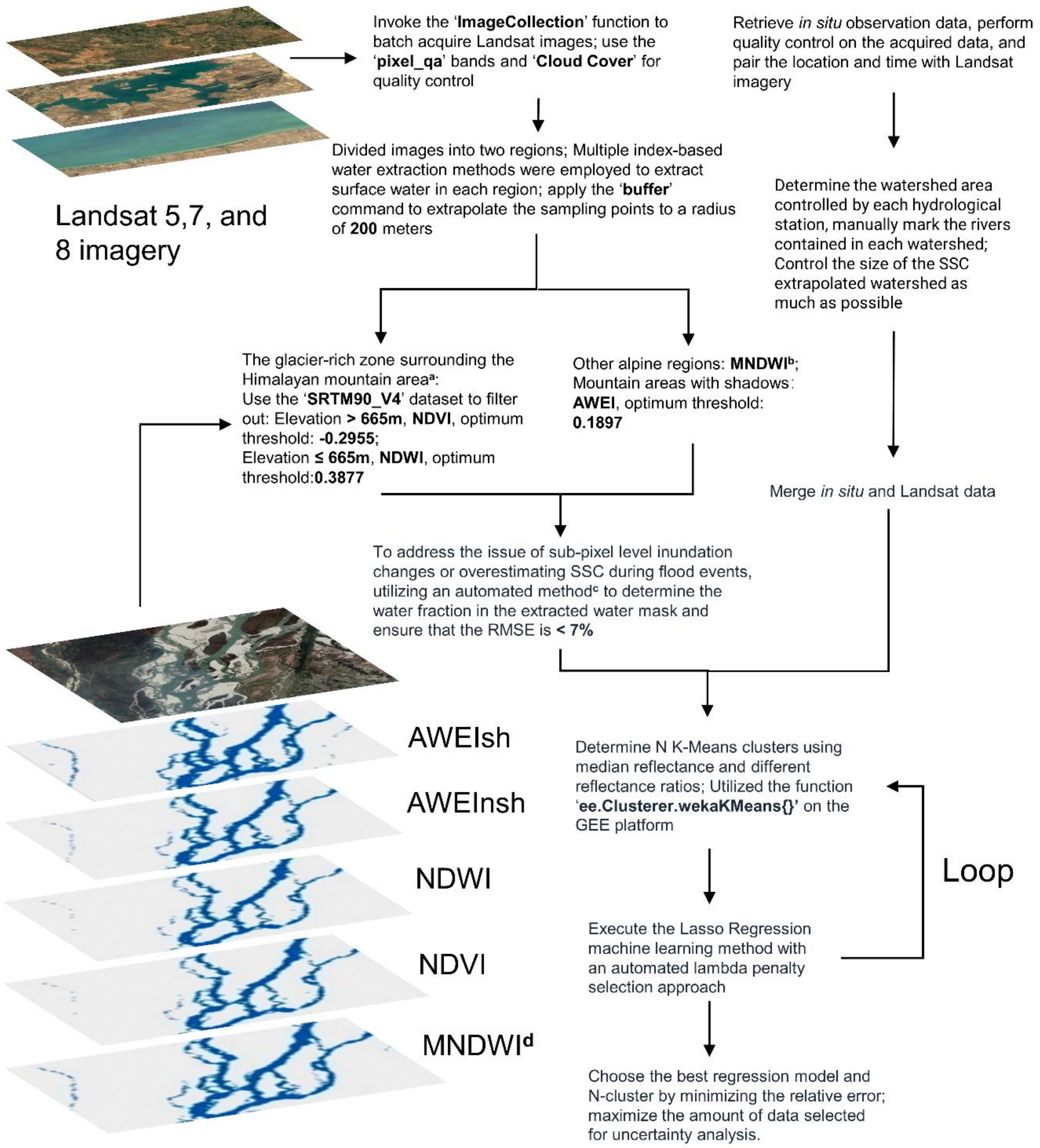


**Supplementary Fig. 11.** The workflow for developing suspended sediment concentration (SSC) extraction and calibration includes preprocessing satellite imagery, applying the multiple index-based water extraction methods in the Tibetan Plateau, integrating satellite and *in situ* data, and testing the application of K-Means clustering and Lasso Regression machine learning algorithms. The bold fonts represent the functions, datasets, and thresholds used in Google Earth Engine.

[a] This area includes several major headwater basins in the glacier-rich region along the Himalayas, including the Indus, Ganges, and Brahmaputra basins, as well as inland basins such as the Amu Darya and Tarim basins.

[b] The threshold for MNDWI was referred to ref.[7].

[c] The automatic water extraction method is described in ref.[12]. We debugged this index in GEE and applied it to water bodies in the Tibetan Plateau.

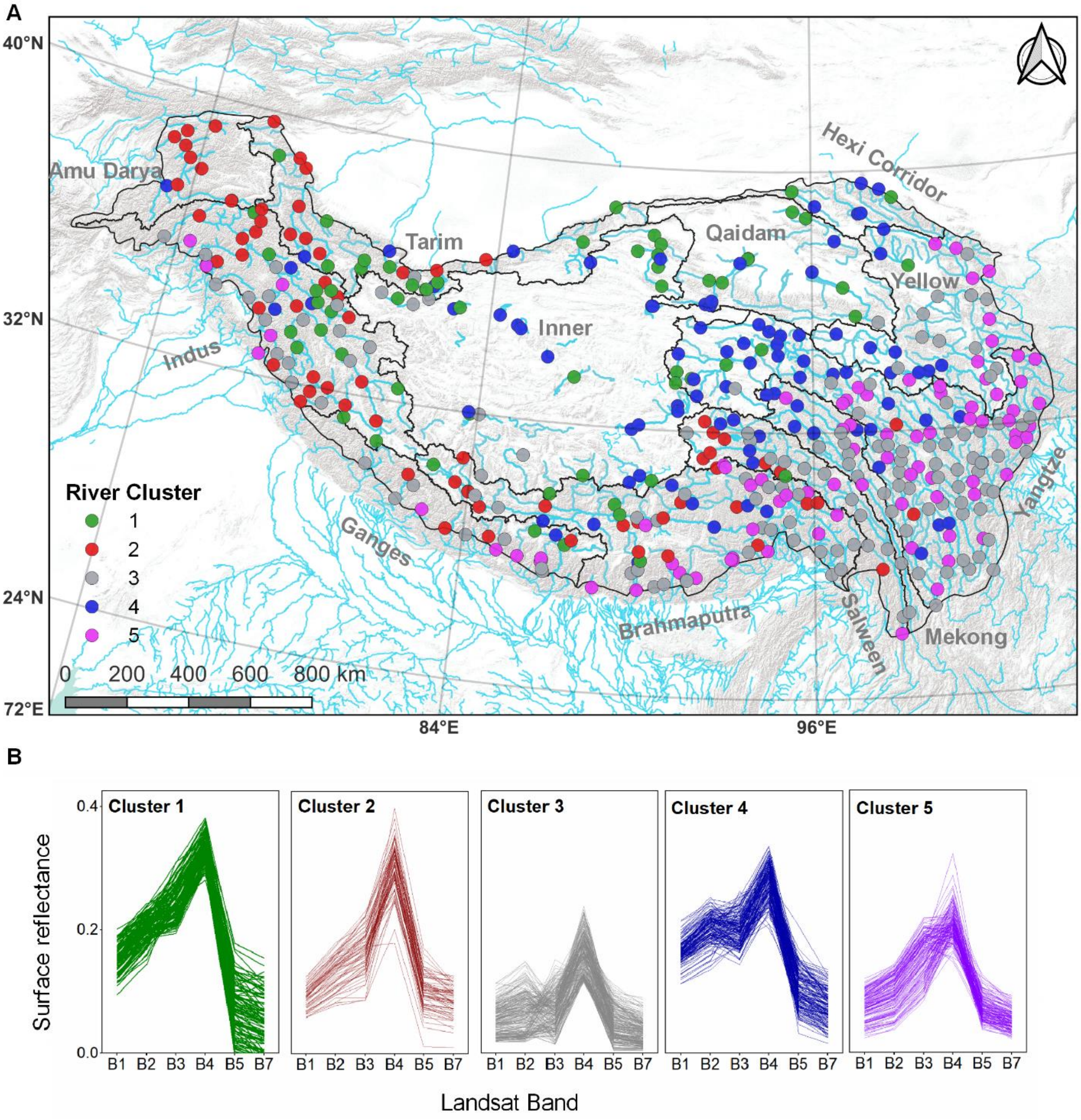


**Supplementary Fig. 12.** K-Means clustering analysis groups rivers into five clusters. (A) A map shows the assigned cluster of each river section (> 90m). The selection of each section is spatially averaged. (B) Parallel line plots show the average surface reflectance in each Landsat 5, 7, and 8 bands for each river in each cluster. Each river is represented by a single line, and colors correspond to those in A. The approach of K-Means clustering is followed by Dethier et al[1]. Base map and inset courtesy of ESRI, USGS, and NOAA.

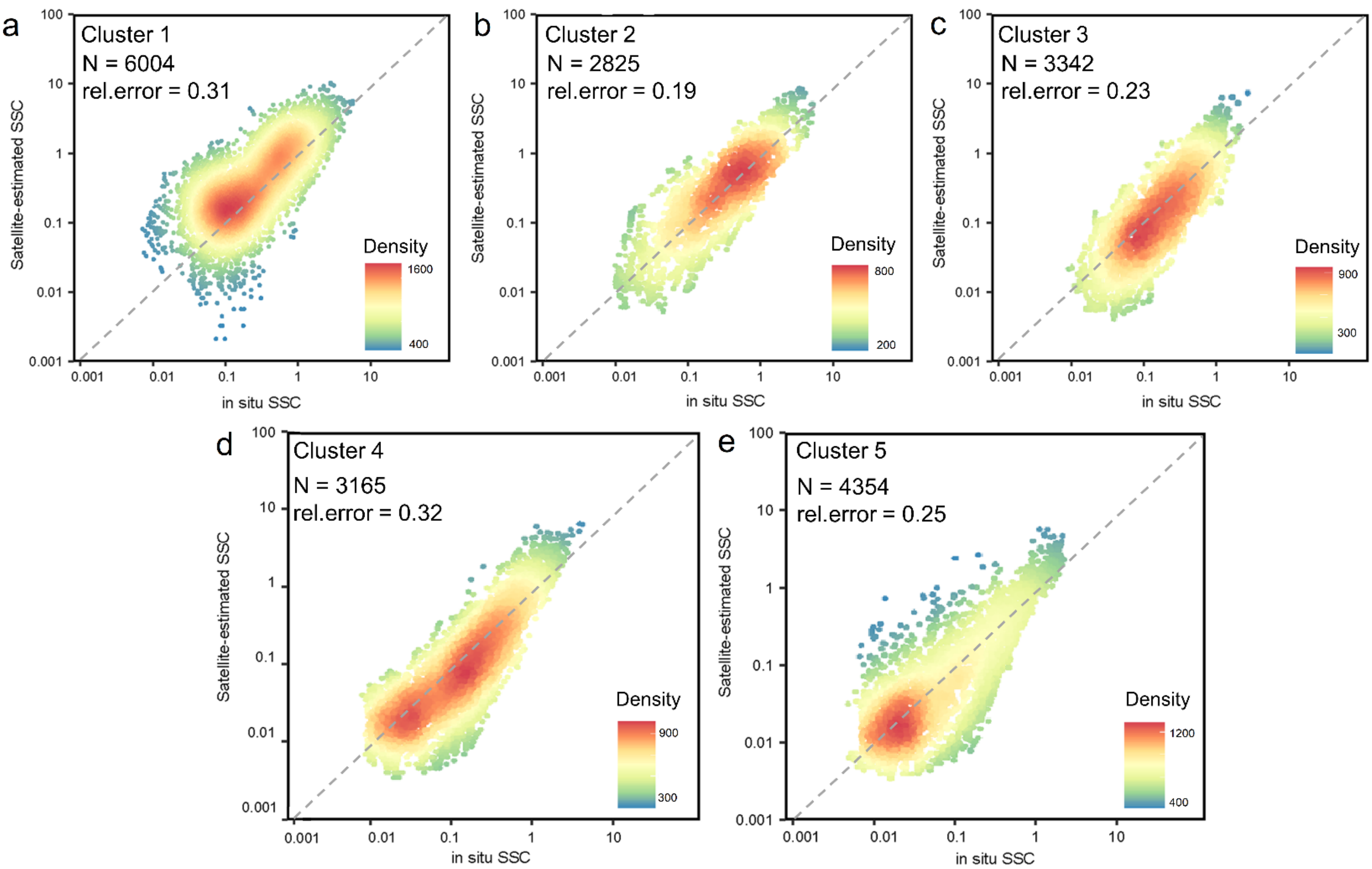


**Supplementary Fig. 13.** Satellite-estimated SSC calibration model outputs (Validation data) were juxtaposed with *in situ* SSC measurements from river sampling sites. Each K-Means cluster grouping corresponds to a calibration model, pairing respectively to the spatial distribution of river optical pixels shown in Fig. S12. We utilized a total of ~19,690 spatially corresponding *in situ* measurements, which also coincide in time with the actual sampling day. The relative error of each model ranges from 0.19 to 0.32, underscoring its stability and robustness over extensive regions.

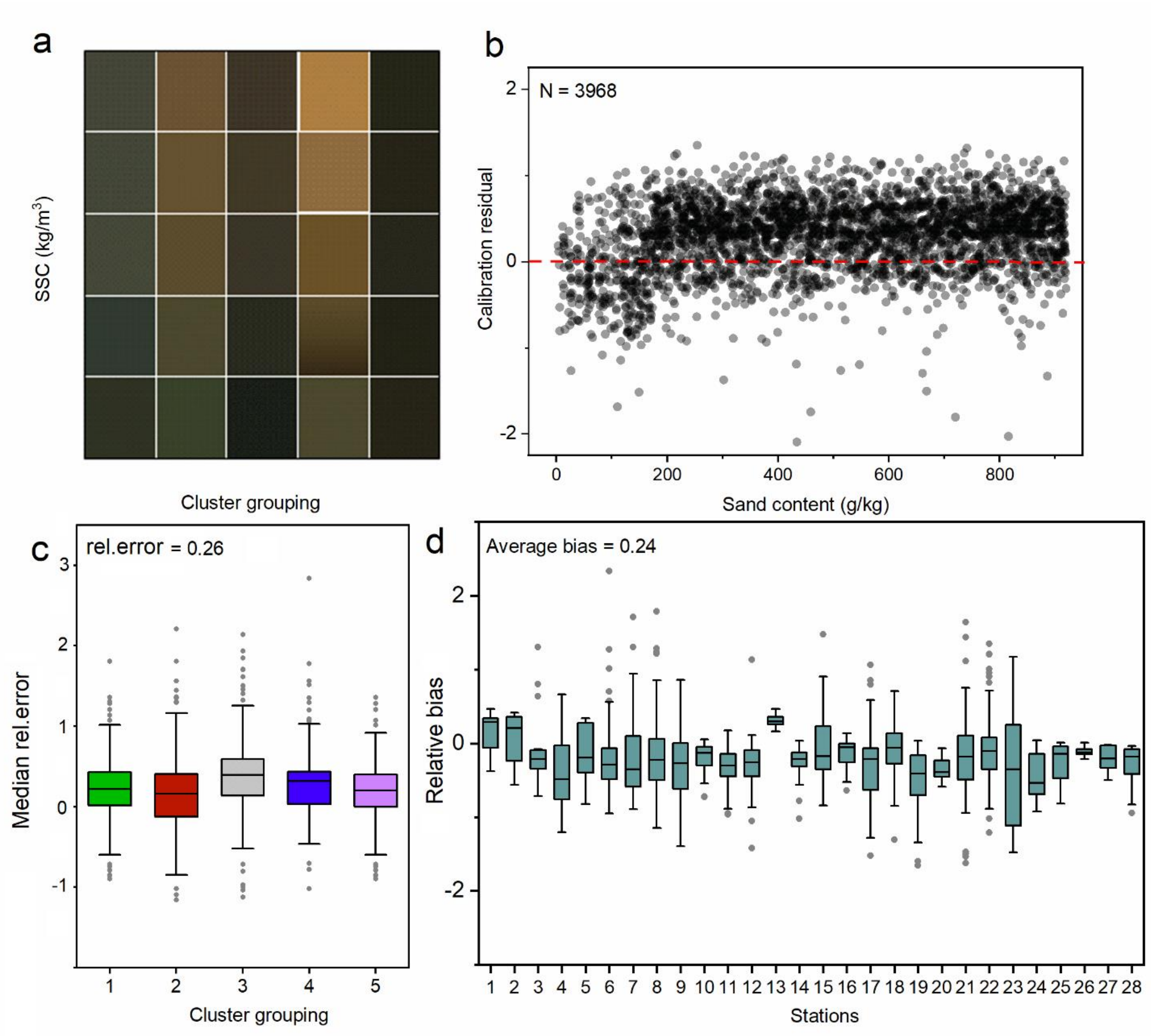


**Supplementary Fig. 14.** (a) The median true color (RGB) representation highlights the SSC gradient for each cluster, which is consistent with Fig. 1. The SSC gradient ranges from 0-0.5, 0.5-2, 2-3, 3-4, and > 4 kg/m$^3$, emphasizing the differences in SSC performance for different groups of rivers. (b) The calibration model shows that as the sand content increases, the estimated SSC becomes more unbiased. However, when the sand content is low, the SSC may be underestimated, but the calibration residual remains within +/-1. (c) The relative error of the average model for the five clusters corresponding to the clustering method is shown, with colors consistent with the clustering classification. (d) The median relative bias between the satellite-estimated SSC and the measured data from the corresponding 28 stations is shown, with the relative bias being 0.24.

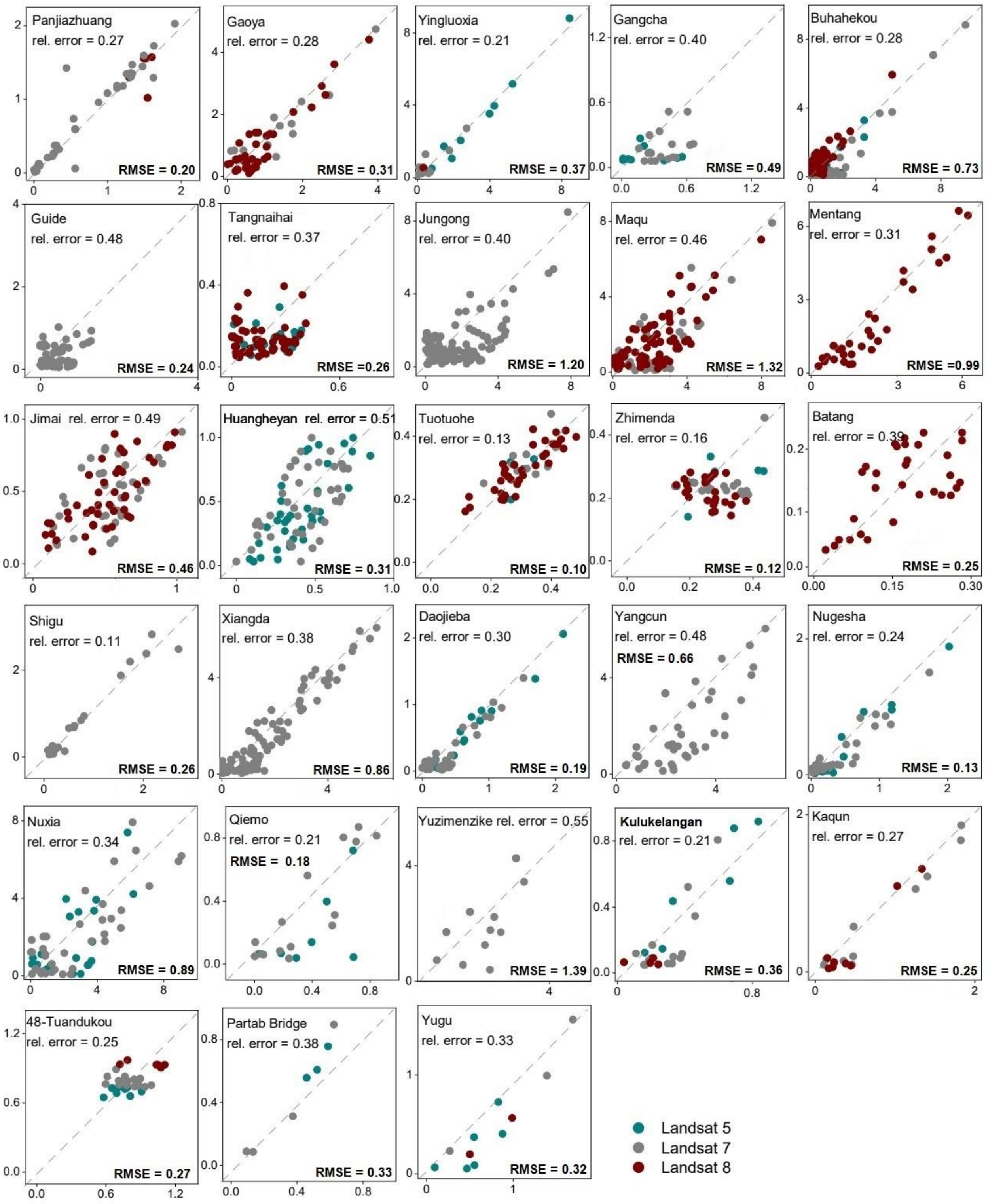


**Supplementary Fig. 15.** Validation results between satellite-estimate SSCs and *in situ* SSCs that were sourced from actual measurement records of 28 hydrological stations. The relative error and root-mean-square error (RMSE) is also counted, indicating the robustness of the algorithm.

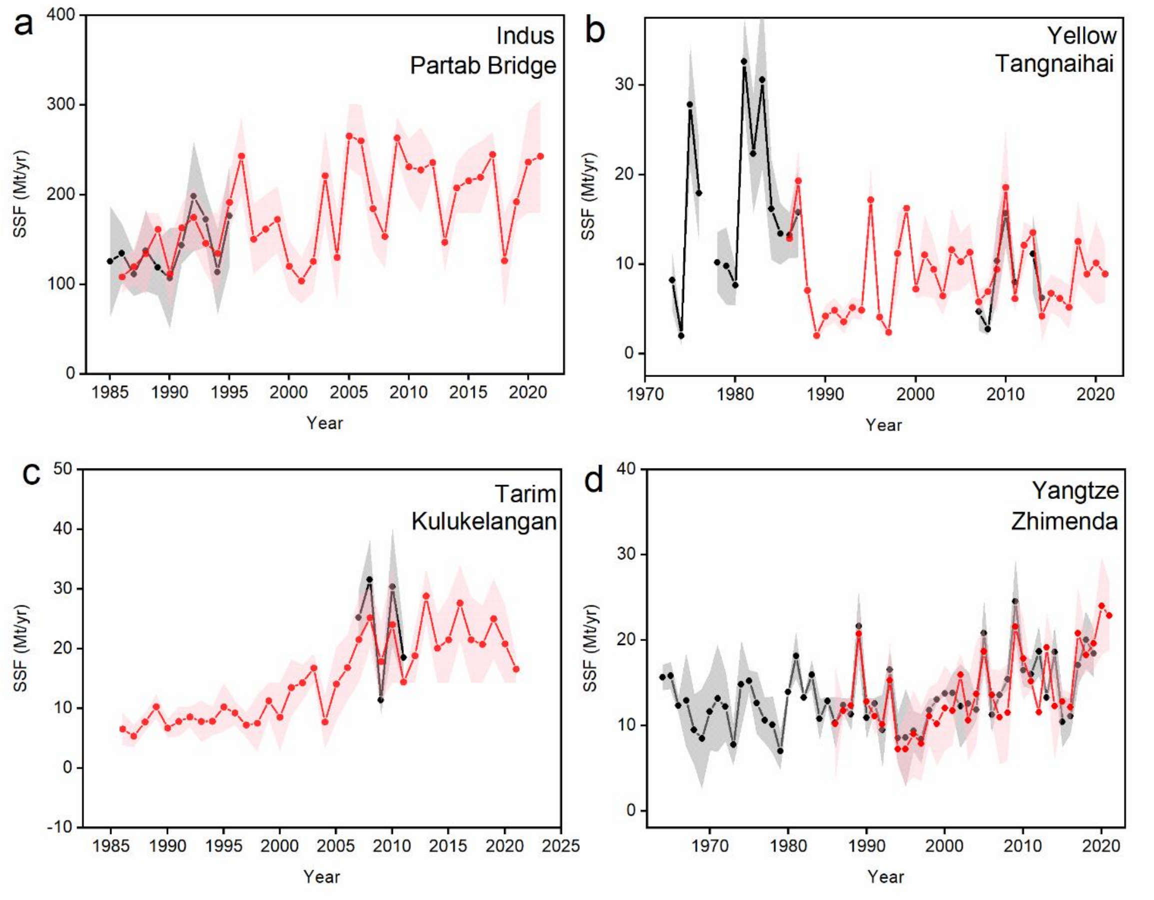


**Supplementary Fig. 16.** The comparison plots depict the suspended sediment flux (Mt/yr) estimated using in situ methods (black) and the satellite-based methods employed in this study (red). The in situ data used in this comparison were obtained from various sources, including (A) data collected from the upper Indus River as reported in ref[35], (B) in situ measurements from the Yellow River (China), (C) data collected from the Tarim River (China) by the Ministry of Water Resources of China and reported in ref[36], and (D) in situ measurements from the Yangtze River (China) collected by the Ministry of Water Resources of China.

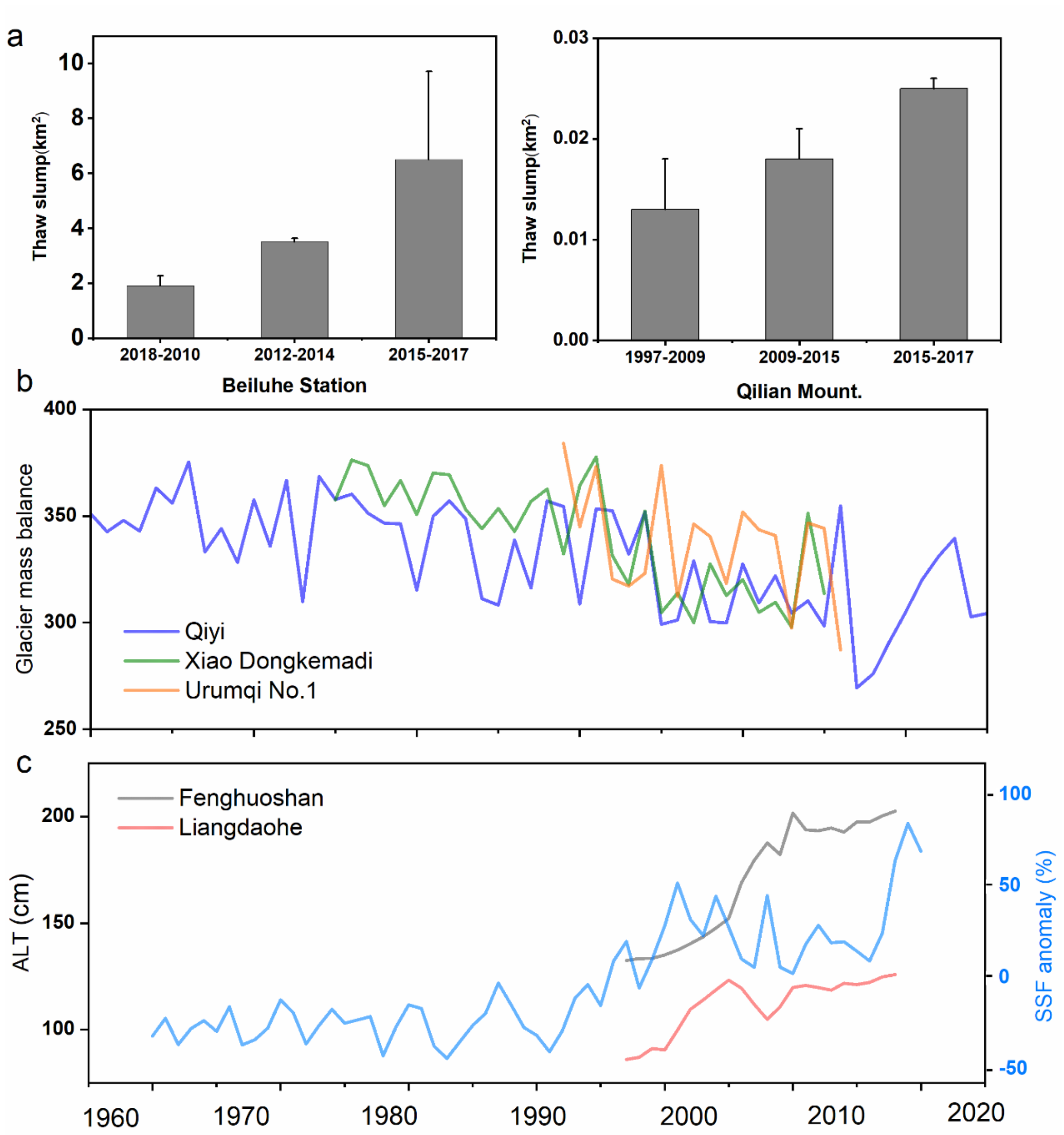


**Supplementary Fig. 17.** Satellite-estimated sediment flux in response to changes in the shifts in cryospheric elements. (a) Marked expansions in the thaw slump area over recent years were observed at Beiluhe and Qilian Mountain stations (upstream regions). (b) Annual glacier mass balances (in mm water equivalent) observed from three regions: Qiyi Glacier (blue line) resides close to S2 (Gaoya) and S3 (Yingluoxia) stations, Xiao Dongkemadi Glacier (green line) lies adjacent to S13 (Tuotuohe) station, and the Urumqi No.1 Glacier is near the northern Tianshan basin, beyond this study' focus. These data highlight three of the Tibetan Plateau's most prolonged glacier melt rate escalations. (c) A surge in permafrost active layer thickness (ALT) corresponds with suspended sediment flux (SSF) anomalies (expressed in percentages) across the Tibetan Plateau. Accelerated shifts in cryospheric elements, encompassing glaciers, permafrost, and thermokarst landscape expansion, inherently amplify erosion and sediment availability, resulting in a significant increase in sediment flux. The data are sourced from refs[37–39].

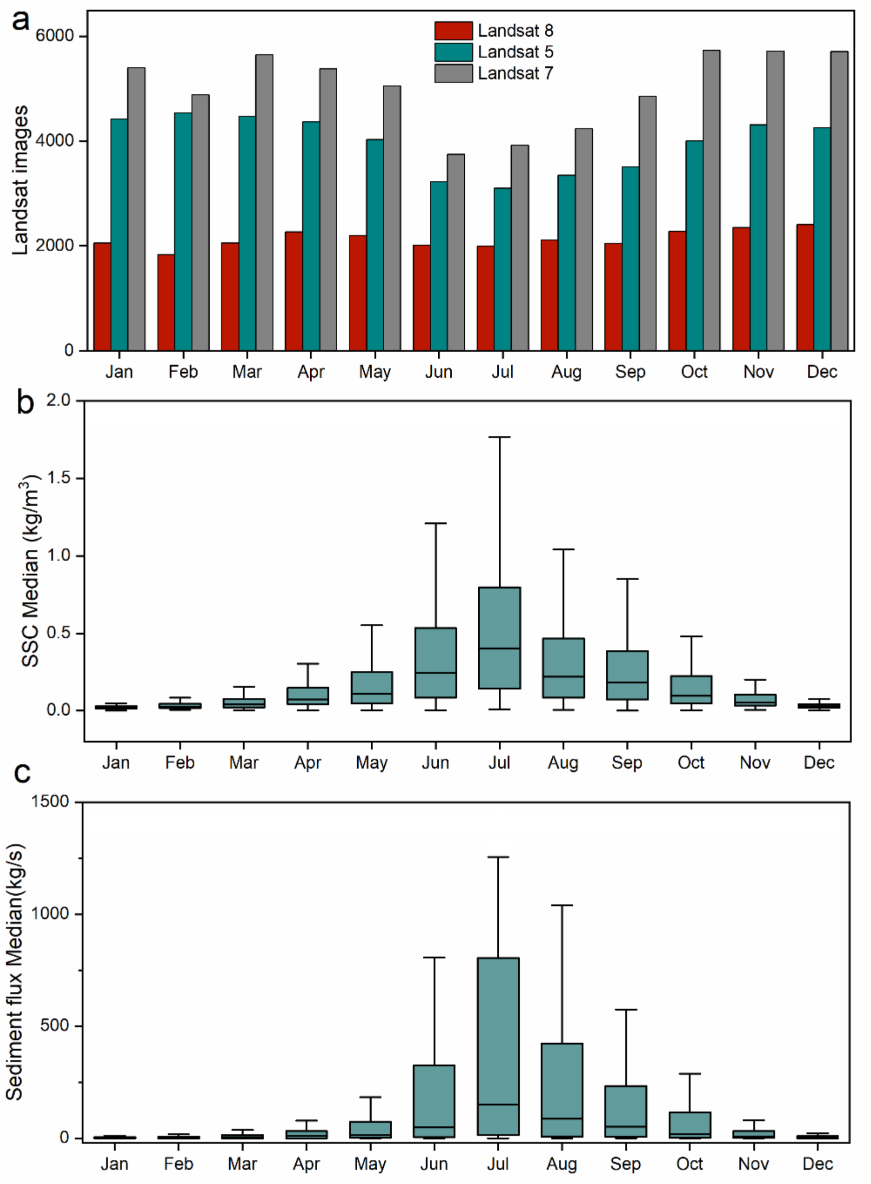


**Supplementary Fig. 18.** The monthly distribution of Landsat images used, as well as the monthly distribution of median SSC and sediment flux extracted by the satellite. The data was calculated on a monthly scale from 1986 to 2021. (a) The monthly distribution of Landsat images (cloud cover < 20%) is shown, and the interannual distribution can be found in Fig. S1C. Stringent quality control measures were implemented to mask out various interferences, such as cloud cover, ice and snow, and mountain shadows, ensuring that non-aqueous pixels had minimal impact on the analysis. (b) and (c) depict the annual distributions of satellite-estimated SSCs and sediment flux. On a broader scale encompassing the Tibetan Plateau, SSC and sediment flux are predominantly concentrated during the monsoon season (summer)[37]. While many rivers in permafrost and glacier regions become dormant during the surface freeze in winter[40,41] (such as the Indus River, the upper reaches of the Yangtze above the Tuotuo station, and the Ganges River), there are also some rivers (mostly in the eastern and southern regions of the Tibetan Plateau) that remain ice-free throughout the year, resulting in a continued albeit relatively lower sediment discharge during the winter[40].

**Supplementary Table. 1.** A summary of the headwater basins and their sampling information.

| No. | Stations[a] | Lon. | Lat. | Headwater | Basin | Area (km$^2$) | Counts | Data accessible period[b] |
|---|---|---|---|---|---|---|---|---|
| S1 | Panjiazhuang | 96.52 | 40.55 | Shule R. | Shule R. | 18496 | 45 | 2015-2019 |
| S2 | Gaoya | 100.39 | 39.14 | Hei R. | Hei R. | 20299 | 53 | 2006-2012 |
| S3 | Yingluoxia | 100.18 | 38.80 | Hei R. | Hei R. | 10009 | 22 | 2006-2007 |
| S4 | Gangcha | 100.13 | 37.32 | Yikewulan R. | Qinghai Lake | 1400 | 39 | 2006-2007 |
| S5 | Buhahekou | 99.74 | 37.04 | Buha R. | Qinghai Lake | 14300 | 172 | 2006-2016 |
| S6 | Guide | 101.40 | 36.03 | Yellow R. | Yellow R. | 13650 | 364 | 1985 |
| S7 | Tangnaihai | 100.15 | 35.50 | Yellow R. | Yellow R. | 120900 | 3668 | 1985-1987;2007-2014 |
| S8 | Jungong | 100.65 | 34.69 | Yellow R. | Yellow R. | 5183 | 3133 | 2008-2013 |
| S9 | Maqu | 102.08 | 33.96 | Yellow R. | Yellow R. | 86048 | 2601 | 1985-2014 |
| S10 | Mentang | 101.05 | 33.77 | Yellow R. | Yellow R. | 3363 | 1140 | 2008-2013 |
| S11 | Jimai | 99.65 | 33.77 | Yellow R. | Yellow R. | 45868 | 2397 | 1985-1987;2007-2014 |
| S12 | Huangheyan | 98.10 | 34.53 | Yellow R. | Yellow R. | 21480 | 8412 | 1985-2007 |
| S13 | Tuotuohe | 92.44 | 34.22 | Tuotuo R. | Yangtze R. | 18200 | 6440 | 1985-2019 |
| S14 | Zhimenda | 97.24 | 33.01 | Tongtian R. | Yangtze R. | 137732 | 12782 | 1985-2019 |
| S15 | Batang | 99.01 | 29.77 | Jinsha R. | Yangtze R. | 187873 | 35 | 2006-2007 |
| S16 | Shigu | 99.95 | 26.91 | Jinsha R. | Yangtze R. | 214200 | 96 | 2006-2007 |
| S17 | Xiangda | 96.48 | 32.25 | Zhaqu R. | Mekong R. | 17907 | 214 | 1985 |
| S18 | Daojieba | 98.88 | 24.98 | Salween R. | Salween R. | 124800 | 113 | 2007-2009 |
| S19 | Yangcun | 91.82 | 29.27 | YarlungTsangpo R. | Brahmaputra R. | 153191 | 43 | 2007-2009 |
| S20 | Nugesha | 89.71 | 29.32 | YarlungTsangpo R. | Brahmaputra R. | 106378 | 136 | 2007-2009 |
| S21 | Nuxia | 94.65 | 29.47 | YarlungTsangpo R. | Brahmaputra R. | 191235 | 219 | 2007-2010 |
| S22 | Qiemo | 85.54 | 38.13 | Cheerchen R. | Tarim R. | 26822 | 349 | 1985-1987;2001-2011 |
| S23 | Yuzimenleke | 77.12 | 37.38 | Tizinafu R. | Tarim R. | 5389 | 23 | 2007 |
| S24 | Kulukelangan | 76.22 | 37.33 | Yarkent R. | Tarim R. | 32880 | 101 | 2007-2011 |
| S25 | Kaqun | 76.90 | 37.98 | Yarkent R. | Tarim R. | 50200 | 111 | 2007-2011 |
| S26 | 48-Tuandukou | 78.33 | 39.43 | Yarkent R. | Tarim R. | 27833 | 77 | 2007-2011 |
| S27 | Partab Bridge | 74.62 | 35.73 | Indus R. | Indus R. | 143130 | 492 | 1985-1995 |
| S28 | Yugo | 76.10 | 35.18 | Shyok R. | Indus R. | 33670 | 265 | 1985-1998 |
| Total | | | | | | | 43542 | |

[a] We emphasizes the spatial monitoring gap in the TP rivers, as less than 30% of them are monitored.

[b] Note that the presented sampling data are dependent on their accessibility to the public. There may be additional *in situ* observations than are not included in this study, such as Station S1, which has provided consistent observations since the 1960s. However, we were only able to collect public records from 2015 to 2019 at S1 due to limited regional policies.

**Supplementary Table. 2.** Comparison with previous studies regarding the sediment concentrations and fluxes in rivers on the Tibetan Plateau.

| River | Basin | Basin area | Glacier area ratio | Permafrost area ratio | Average annual runoff | Satellite-eastimated SSC[c] | Relative bias[d] | Satellite-eastimated SSF | Relative bias[d] | Sources | Time period[e] |
|---|---|---|---|---|---|---|---|---|---|---|---|
| | | ($km^2$) | (%) | (%) | ($km^3$/yr) | (kg/$m^3$) | (%) | (Mt/yr) | (%) | | |
| Yarkent R. | Tarim | 190,669 | 9.62 | 60.23 | 6.01 | 4.06 | 14.22 | 24.40 | 32.15 | ref[36,37] | 1969-2015 |
| Shule R.* | Hexi C. | 61,699 | 3.73 | 80.79 | 1.49 | 3.46 | 16.65 | 4.21 | 21.7 | ref[22,42] | 1957-2017 |
| Babao R.[a] * | Hei R. | 2,452 | 0.44 | 64.52 | 0.77 | 1.06 | 24.34 | 0.85 | 32 | ref[37] | 1968-2017 |
| Yellow R.* | Yellow | 254,191 | 0.13 | 28.90 | 4.11 | 0.70 | 40.15 | 2.88 | 42.13 | ref[37,43] | 1956-2015 |
| Tuotuo R.- upper Jinsha R.[b] * | Yangtze | 477,821 | 0.05 | 30.75 | 23.45 | 0.49 | 16.70 | 11.72 | 24.41 | ref[37] | 1966-2017 |
| Salween R.* | Salween | 130,172 | 1.29 | 30.56 | 54.15 | 0.69 | 7.81 | 37.9 | 9.01 | ref[37,42] | 1964-2011 |
| Zhaqu R. * | Mekong | 90,441 | 0.2 | 56.32 | 4.24 | 0.87 | 36.35 | 5.03 | 36.31 | ref[37,42] | 1963-2017 |
| Yarlung Tsangpo R.* | Brahmaputra | 347,506 | 10.7 | 38.65 | 31.03 | 0.56 | 17.28 | 20.38 | 16.65 | ref[37,42] | 1964-2010 |
| Upper Indus R. | Indus | 319,123 | 27.61 | 53.62 | 34.96 | 6.80 | 21.19 | 114.99 | 12.13 | ref[35,37] | 1983-2008 |
| Amu R. | Amu Darya | 125,633 | 32.13 | 56.19 | 18.65 | 1.91 | 43.52 | 16.97 | 28.71 | ref[44] | 1985-2005 |

| | | | | | | | | | | |
|---|---|---|---|---|---|---|---|---|---|---|
| Ganges R. | Ganges | 121,321 | 30.14 | 45.21 | 28.15 | 2.36 | 28.95 | 4.13 | 46.85 | ref[37] | 1983, 2004 |

[a] In order to demonstrate differences, we also included the relevant information of some small watersheds, such as Babao R. However, it should be noted that their areas were not mentioned in the text due to their relatively small size. The text only discusses the sediment changes in the major rivers on the TP.

[b] The generally accepted definition of the Yangtze River source region refers to the watershed above the Zhimenda basin. For ease of calculation and comparison, we included the overall Upper Yangtze River basin on the TP, including the Jinsha River and Yalong River in this study.

[c] The area and relevant information we compiled were derived from the watershed delineation at a large spatial scale on the plateau, please refer to ref.[45]. Please note that the differences in SSF we calculated were scaled proportionally based on the basin area.

[d] Relative bias as compared to refs. [23,24,25,28,29,30,31]. We calculated the relative bias (%) between satellite-estimate sediment yield or flux and the reference data using the equation $(SSF_{satellite}-SSF_{refs})/SSF_{refs}$.

[e] The corresponding measured sediment record time was obtained from the cited refs above. Note that the study period in this study is from January 1, 1986 to December 31, 2021.

* The river sediment samples marked with an asterisk in superscript were measured using daily depth-integration techniques, and the corresponding sampling stations can be found in Table S1. However, for other rivers, especially international rivers like the Indus and Amu Darya, the original references did not specify their sampling methods.

**Supplementary Table. 3.** Satellite-derived dynamics and deposition proportions of major mainstreams in primary headwater areas.

| Basins | Satellite-estimated sediment yield | Runoff increase rate [a] | SSC increase rate | SSF increase rate | *in situ* SSF [a] | Sediment deposition ratio [b] | | |
|---|---|---|---|---|---|---|---|---|
| | (t/km$^2$/yr) | (%/10yr) | (%/10yr) | (%/10yr) | Mt/yr | Upstream (%) | Midstream (%) | Downstream (%) |
| Tarim | 759.92 | 3.64 | 6.27 | 12.53 | 7.8 | 15.03 | 19.32 | n/a [c] |
| Hexi C. | 298.23 | 3.95 | 0.63 | 1.24 | 3.46 | 8.64 | 32.59 | n/a |
| Yellow | 30.67 | 4.12 | 2.07 | 4.35 | 2.02 | 5.89 | 46.87 | 13.49 |
| Yangtze | 64.52 | 8.09 | 7.5 | 15.21 | 9.42 | 10.24 | 21.34 | 14.25 |
| Salween | 137.55 | 12.15 | 10.16 | 18.36 | 34.77 | 24.32 | 17.46 | 2.15 |
| Mekong | 355.6 | 4.24 | 3.45 | 6.89 | 3.69 | 15.73 | 7.31 | 1.07 |
| Brahmaputra | 124.11 | 7.03 | 4.21 | 9.46 | 17.47 | 4.75 | 45.29 | 5.48 |
| Indus | 891.69 | 6.55 | 8.21 | 17.87 | 111.47 | 33.94 | 41.67 | 11.23 |
| Ganges | 334.07 | 3.31 | 6.45 | 11.27 | 14.93 | 31.25 | n/a | n/a |
| Amu Darya | 229.83 | 2.25 | 11.2 | 24.57 | 3.18 | 32.64 | n/a | n/a |

[a] The period for evaluating the increase rate spans from 1986 to 2021; however, please note that these dates may differ from those cited in previous references. The records of sediment *in situ* observation were obtained from existing hydrological stations as well as through statistical analysis of data provided by refs.[23,24,25,28,29,30,31].

[b] The sediment deposition ratio is a metric calculated by determining the ratio between the regional sediment deposition amount, calculated through the sediment balance model, and the sediment flux reported by the watershed outlet using the equation $(SSF_{deposition}-SSF_{outlet})/SSF_{outlet}$.

[c] The upper, middle, and lower reaches of the watershed are partitioned into thirds based on the length of the primary river within the watershed, which is obtained from MERIT Hydro[46], Global River Widths from Landsat[47]. Note that "n/a" signifies results obtained from processing along the defined TP watershed boundary. For example, in the case of the Ganges River basin, which is primarily located in the upstream region along the TP boundary, the vast majority of the river falls within the upper region of the defined boundary.

# References


1. Dethier, E. N., Renshaw, C. E. & Magilligan, F. J. Rapid changes to global river suspended sediment flux by humans. *Science* **376**, 1447–1452 (2022).
2. Overeem, I. *et al.* Substantial export of suspended sediment to the global oceans from glacial erosion in Greenland. *Nature Geosci* **10**, 859–863 (2017).
3. Kilham, N. E., Roberts, D. & Singer, M. B. Remote sensing of suspended sediment concentration during turbid flood conditions on the Feather River, California-A modeling approach: A UNIVERSAL MODEL OF SUSPENDED SEDIMENT CONCENTRATION. *Water Resour. Res.* **48**, (2012).
4. Pavelsky, T. M. & Smith, L. C. Remote sensing of suspended sediment concentration, flow velocity, and lake recharge in the Peace-Athabasca Delta, Canada: REMOTE SENSING OF SUSPENDED SEDIMENT CONCENTRATION. *Water Resour. Res.* **45**, (2009).
5. Sheffield, J. *et al.* Satellite Remote Sensing for Water Resources Management: Potential for Supporting Sustainable Development in Data-Poor Regions. *Water Resour. Res.* **54**, 9724–9758 (2018).
6. Long, C. M. & Pavelsky, T. M. Remote sensing of suspended sediment concentration and hydrologic connectivity in a complex wetland environment. *Remote Sensing of Environment* **129**, 197–209 (2013).
7. Dethier, E. N., Renshaw, C. E. & Magilligan, F. J. Toward Improved Accuracy of Remote Sensing Approaches for Quantifying Suspended Sediment: Implications for Suspended-Sediment Monitoring. *JGR Earth Surface* **125**, (2020).

8. Montanher, O. C., Novo, E. M. L. M., Barbosa, C. C. F., Rennó, C. D. & Silva, T. S. F. Empirical models for estimating the suspended sediment concentration in Amazonian white water rivers using Landsat 5/TM. *International Journal of Applied Earth Observation and Geoinformation* **29**, 67–77 (2014).
9. Martinez, J.-M., Espinoza-Villar, R., Armijos, E. & Silva Moreira, L. The optical properties of river and floodplain waters in the Amazon River Basin: Implications for satellite-based measurements of suspended particulate matter: AMAZON RIVER WATER OPTICAL PROPERTIES. *J. Geophys. Res. Earth Surf.* **120**, 1274–1287 (2015).
10. Hudson, B. *et al.* MODIS observed increase in duration and spatial extent of sediment plumes in Greenland fjords. *The Cryosphere* **8**, 1161–1176 (2014).
11. Acharya, T., Subedi, A. & Lee, D. Evaluation of Water Indices for Surface Water Extraction in a Landsat 8 Scene of Nepal. *Sensors* **18**, 2580 (2018).
12. Liang, J. & Liu, D. Automated estimation of daily surface water fraction from MODIS and Landsat images using Gaussian process regression. *International Journal of Remote Sensing* **42**, 4261–4283 (2021).
13. Walling, D. E. & Fang, D. Recent trends in the suspended sediment loads of the world's rivers. *Global and Planetary Change* **39**, 111–126 (2003).
14. Tian, Q. *et al.* Declining Sediment Discharge in the Yangtze River From 1956 to 2017: Spatial and Temporal Changes and Their Causes. *Water Res* **57**, (2021).
15. Najafi, S., Dragovich, D., Heckmann, T. & Sadeghi, S. H. Sediment connectivity concepts and approaches. *CATENA* **196**, 104880 (2021).

16. Syvitski, J. *et al.* Earth's sediment cycle during the Anthropocene. *Nat Rev Earth Environ* **3**, 179–196 (2022).
17. Zhang, T. *et al.* Warming-driven erosion and sediment transport in cold regions. *Nat Rev Earth Environ* **3**, 832–851 (2022).
18. Feng, D. *et al.* Recent changes to Arctic river discharge. *Nat Commun* **12**, 6917 (2021).
19. Balasubramanian, S. V. *et al.* Robust algorithm for estimating total suspended solids (TSS) in inland and nearshore coastal waters. *Remote Sensing of Environment* **246**, 111768 (2020).
20. Hilger, L. & Beylich, A. A. Sediment Budgets in High-Mountain Areas: Review and Challenges. in *Geomorphology of Proglacial Systems* (eds. Heckmann, T. & Morche, D.) 251–269 (Springer International Publishing, 2019). doi:10.1007/978-3-319-94184-4_15.
21. Gorelick, N. *et al.* Google Earth Engine: Planetary-scale geospatial analysis for everyone. *Remote Sensing of Environment* **202**, 18–27 (2017).
22. Wang, S., Zhao, Q. & Pu, T. Assessment of water stress level about global glacier-covered arid areas: A case study in the Shule River Basin, northwestern China. *Journal of Hydrology: Regional Studies* **37**, 100895 (2021).
23. Yan, D., Ma, N. & Zhang, Y. Development of a fine-resolution snow depth product based on the snow cover probability for the Tibetan Plateau: Validation and spatial–temporal analyses. *Journal of Hydrology* **604**, 127027 (2022).

24. Smith, T. & Bookhagen, B. Changes in seasonal snow water equivalent distribution in High Mountain Asia (1987 to 2009). *Sci. Adv.* **4**, e1701550 (2018).

25. Liu, F. *et al.* High-resolution and three-dimensional mapping of soil texture of China. *Geoderma* **361**, 114061 (2020).

26. Yan, X., Zhou, Y. & Yao, S. Landform and Characteristics of Flow and Sediment of Rivers in Source Region of Yangtze River. *Journal of Yangtze River Scientific Research Institute (in Chinese)* **36**, 10–15 (2019).

27. Zhang, X. *et al.* GLC_FCS30: global land-cover product with fine classification system at 30 m using time-series Landsat imagery. *Earth Syst. Sci. Data* **13**, 2753–2776 (2021).

28. Wang, T., Yang, D., Zheng, G. & Shi, R. Possible negative effects of earlier thaw onset and longer thaw duration on vegetation greenness over the Tibetan Plateau. *Agricultural and Forest Meteorology* **326**, 109192 (2022).

29. Hersbach, H. *et al.* The ERA5 global reanalysis. *Q.J.R. Meteorol. Soc.* **146**, 1999–2049 (2020).

30. Li, D. *et al.* High Mountain Asia hydropower systems threatened by climate-driven landscape instability. *Nat. Geosci.* **15**, 520–530 (2022).

31. Zheng, G. *et al.* Increasing risk of glacial lake outburst floods from future Third Pole deglaciation. *Nat. Clim. Chang.* **11**, 411–417 (2021).

32. Mulligan, M., van Soesbergen, A. & Sáenz, L. OPEN GOODD, a global dataset of more Data Descriptor than 38,000 georeferenced dams. *Scientific Data*.

33. Raup, B. *et al.* The GLIMS geospatial glacier database: A new tool for studying glacier change. *Global and Planetary Change* **56**, 101–110 (2007).

34. Zhao, L. *et al.* Changing climate and the permafrost environment on the Qinghai–Tibet (Xizang) plateau. *Permafrost and Periglac Process* **31**, 396–405 (2020).

35. Ali, K. F. & De Boer, D. H. Spatial patterns and variation of suspended sediment yield in the upper Indus River basin, northern Pakistan. *Journal of Hydrology* **334**, 368–387 (2007).

36. Deng, S. Y. Analysis for the Characteristics of Sediment in Tarim River. *AMM* **641–642**, 25–28 (2014).

37. Li, D. *et al.* Exceptional increases in fluvial sediment fluxes in a warmer and wetter High Mountain Asia. *Science* **374**, 599–603 (2021).

38. Yao, T. *et al.* Different glacier status with atmospheric circulations in Tibetan Plateau and surroundings. *Nature Clim Change* **2**, 663–667 (2012).

39. Luo, J., Niu, F., Lin, Z., Liu, M. & Yin, G. Recent acceleration of thaw slumping in permafrost terrain of Qinghai-Tibet Plateau: An example from the Beiluhe Region. *Geomorphology* **341**, 79–85 (2019).

40. Li, J. *et al.* Impacts of climate change and freeze–thaw cycles on water and sediment fluxes in the headwater region of the Yangtze River, Qinghai–Tibet Plateau. *CATENA* **227**, 107112 (2023).

41. Li, D., Li, Z., Zhou, Y. & Lu, X. Substantial Increases in the Water and Sediment Fluxes in the Headwater Region of the Tibetan Plateau in Response to Global Warming. *Geophys. Res. Lett.* **47**, (2020).

42. Zhang, F., Zeng, C., Wang, G., Wang, L. & Shi, X. Runoff and sediment yield in relation to precipitation, temperature and glaciers on the Tibetan Plateau. *International Soil and Water Conservation Research* **10**, 197–207 (2022).

43. Shi, X. *et al.* Spatiotemporal variations of suspended sediment transport in the upstream and midstream of the Yarlung Tsangpo River (the upper Brahmaputra), China: Spatiotemporal variations of sediment in Yarlung Tsangpo River. *Earth Surf. Process. Landforms* **43**, 432–443 (2018).

44. Ikramova, M. Estimation of sediment loads: the Tuyamuyun reservoir on Amudarya river. *ESR* 199–202 (2016) doi:10.20534/ESR-16-7.8-199-202.

45. Zhao, L. *et al.* A synthesis dataset of permafrost thermal state for the Qinghai–Tibet (Xizang) Plateau, China. *Earth Syst. Sci. Data* **13**, 4207–4218 (2021).

46. Yamazaki, D. *et al.* MERIT Hydro: A High-Resolution Global Hydrography Map Based on Latest Topography Dataset. *Water Resources Research* **55**, 5053–5073 (2019).

47. Allen, G. H. & Pavelsky, T. M. Global extent of rivers and streams. *Science* **361**, 585–588 (2018).
