# Supplementary material for: Recent intensified erosion and massive sediment deposition in Tibetan Plateau rivers
Source: Nat Commun. 2024 Jan 24;15:722. doi: 10.1038/s41467-024-44982-0 (PMC10808212; doi:10.1038/s41467-024-44982-0)
Supplement: Supplementary file 3 — Inventory of Supporting Information [file 41467_2024_44982_MOESM3_ESM.docx]

Inventory of Supporting Information

**Manuscript #: NCOMMS-23-17241A.**

| Please complete each of the Inventory Tables below to outline your Extended Data and Supplementary Information items.  There are four sections:   - *Extended Data* - *Supplementary Information: PDF Files* - *Supplementary Information: Additional Files* - *Source Data*   Each section includes specific instructions. Please complete these tables as fully as possible. We ask that you avoid using spaces in your file names, and instead use underscores, i.e.: Smith_ED_Fig1.jpg not Smith ED Fig1.jpg  Please note that titles and descriptive captions will only be lightly edited, so please ensure that you are satisfied with these prior to submission.  If you have any questions about any of the information contained in this inventory, please contact the journal. |
| --- |
|  |

**Corresponding author name(s):** Genxu Wang and Chunlin Song.

1. **Extended Data**

**We have no Extended Data in this paper.**

1. **Supplementary Information:**
2. **PDF Files**

**We have uploaded Supplementary information in the form of a PDF. The Reporting Summary has been included in the Cover Letter as per the editor's instructions.**

**Complete the Inventory below for all additional textual information and any additional Supplementary Figures, which should be supplied in one combined PDF file.**

- **Row 1:** A combined PDF containing any Supplementary Text, Discussion, Notes, Additional Supplementary Figures, Supplementary Protocols, simple tables, and all associated legends. **Only one such file is permitted**.

**Done.**

- **Row 2:** Nature Research’s Reporting Summary; if previously requested by the editor, please provide an updated Summary, fully completed, without any mark-ups or comments. **(Reporting Summaries are not required for all manuscripts.)**

**Summary：Climate change intensifies erosion and sediment transport in rivers of the**

**Tibetan Plateau. Satellite data unveils unprecedented patterns of sediment deposition in**

**rivers. Pronounced spatiotemporal heterogeneities within and across basins are found.**

**Note:** Please do not include a title page within your Supplementary Information file - a cover sheet that includes the title of your paper and a hyperlink to it will be automatically added while preparing your manuscript for publication

| Item | Present? | Filename  Whole original file name including extension. i.e.: Smith_SI.pdf. The extension must be .pdf | A brief, numerical description of file contents.  i.e.: *Supplementary Figures 1-4, Supplementary Discussion, and Supplementary Tables 1-4.* |
| --- | --- | --- | --- |
| Supplementary Information | Yes | Supplementary Information.pdf | Supplementary Discussion; Supplementary Figures 1-18; Supplementary Tables 1-3; |
| Reporting Summary | Yes | Cover Letter |  |
| Peer Review Information | Yes | *OFFICE USE ONLY* |  |

1. **Additional Supplementary Files**

**Complete the Inventory below for all additional Supplementary Files that cannot be submitted as part of the Combined PDF.**

**NONE.**

- Do not list Supplementary Figures in this table (see section 2A)
- Where possible, include the title and description within the file itself
- Spreadsheet-based tables & data should be combined into a workbook with multiple tabs, not submitted as individual files.
- Compressed files are acceptable where necessary. ZIP files are preferred.
- Please note that the *ONLY* allowable types of additional Supplementary Files are:

| - Supplementary Tables | - Supplementary Audio | - Supplementary Videos | - Supplementary Software |
| --- | --- | --- | --- |
| - Supplementary Code | - Supplementary Data, for example: - Source Data for Supplementary Figures - Raw NMR Data, Cryo-EM Data - Computational Data, Crystallographic Data, etc. | | |

| Type | Number  Each type of file (Table, Video, etc.) should be numbered from 1 onwards. Multiple files of the same type should be listed in sequence, i.e.: Supplementary Video 1, Supplementary Video 2, etc. | Filename  Whole original file name including extension. i.e.: *Smith_ Supplementary_Video_1.mov* | Legend or Descriptive Caption  Describe the contents of the file |
| --- | --- | --- | --- |
| Choose an item. |  |  |  |
| Choose an item. |  |  |  |
| Choose an item. |  |  |  |
| Choose an item. |  |  |  |
| Choose an item. |  |  |  |
| Choose an item. |  |  |  |

***Add rows as needed to accommodate the number of files.***

**3. Source Data**

**Done.**

**Complete the Inventory below for all Source Data files.**

- Acceptable types of Source Data for Main Figures and Extended Data Figures or Tables are:
  - Statistical Source Data
    - Plain Text (ASCII, csv, TXT) or Excel formats only
    - Either one file for each relevant Figure, or a single file containing all source data, with clearly named tabs for each Figure/Extended Data Figure item
  - Full-length, unprocessed gels or blots
    - JPG, TIF, or PDF formats only
    - One file for each relevant Figure containing all supporting blots and/or gels, or a single file with clearly labeled gels or blots for each Figure/ED Figure item
- Source Data for Supplementary Figures is not allowed. Instead:
  - Include Unprocessed Gels or Blots for Supplementary Figures as additional Supplementary Figures to the main Supplementary Information PDF file.
  - Include Statistical Source Data for Supplementary Figures as ‘Supplementary Data’ files and list them in section 2B.
  - Please see [this example of Source Data](https://www.nature.com/articles/s41591-019-0505-4) in a publication.

| Parent Figure or Table | Filename  Whole original file name including extension. i.e.: *Smith_SourceData_Fig1.xls,* or *Smith_ Unmodified_Gels_Fig1.pdf* | Data description  i.e.: Unprocessed western Blots and/or gels, Statistical Source Data, etc. |
| --- | --- | --- |
| Source Data Fig. 1 |  |  |
| Source Data Fig. 2 |  |  |
| Source Data Fig. 3 |  |  |
| Source Data Fig. 4 |  |  |
| Source Data Fig. 5 |  |  |
| Source Data Fig. 6 |  |  |
| Source Data Fig. 7 |  |  |
| Source Data Fig. 8 |  |  |
| Source Data Extended Data Fig./Table 1 |  |  |
| Source Data Extended Data Fig./Table 2 |  |  |
| Source Data Extended Data Fig./Table 3 |  |  |
| Source Data Extended Data Fig./Table 4 |  |  |
| Source Data Extended Data Fig./Table 5 |  |  |
| Source Data Extended Data Fig./Table 6 |  |  |
| Source Data Extended Data Fig./Table 7 |  |  |
| Source Data Extended Data Fig./Table 8 |  |  |
| Source Data Extended Data Fig./Table 9 |  |  |
| Source Data Extended Data Fig./Table 10 |  |  |
